# Role of TGFβ1 and WNT6 in FGF2 and BMP4-driven endothelial differentiation of murine embryonic stem cells

**DOI:** 10.1007/s10456-021-09815-4

**Published:** 2021-09-03

**Authors:** Anna Gualandris, Alessio Noghero, Davide Cora’, Elena Astanina, Marco Arese, Federico Bussolino

**Affiliations:** 1 Department of Oncology, University of Turin, Candiolo, Italy; 2 Candiolo Cancer Institute-FPO-IRCCS, Candiolo, Italy; 3 Lovelace Biomedical Research Institute, Albuquerque, NM USA; 4 Department of Translational Medicine, Piemonte Orientale University, Novara, Italy; 5 Center for Translational Research on Autoimmune and Allergic Disease (CAAD), Novara, Italy

**Keywords:** ES cells, PA6 cells, Endothelial differentiation, FGF2, BMP4, TGFβ1, WNT6

## Abstract

Embryonic stem cells (ES) are a valuable source of endothelial cells. By co-culturing ES cells with the stromal PA6 cells, the endothelial commitment can be achieved by adding exogenous FGF2 or BMP4. In this work, the molecular pathways that direct the differentiation of ES cells toward endothelium in response to FGF2 are evaluated and compared to those activated by BMP4. To this purpose the genes expression profiles of both ES/PA6 co-cultures and of pure cultures of PA6 cells were obtained by microarray technique at different time points. The bioinformatics processing of the data indicated TGFβ1 as the most represented upstream regulator in FGF2-induced endothelial commitment while WNT pathway as the most represented in BMP4-activated endothelial differentiation. Loss of function experiments were performed to validate the importance of TGFβ1 and WNT6 respectively in FGF2 and BMP4-induced endothelial differentiation. The loss of TGFβ1 expression significantly impaired the accomplishment of the endothelial commitment unless exogenous recombinant TGFβ1 was added to the culture medium. Similarly, silencing WNT6 expression partially affected the endothelial differentiation of the ES cells upon BMP4 stimulation. Such dysfunction was recovered by the addition of recombinant WNT6 to the culture medium. The ES/PA6 co-culture system recreates an in vitro complete microenvironment in which endothelial commitment is accomplished in response to alternative signals through different mechanisms. Given the importance of WNT and TGFβ1 in mediating the crosstalk between tumor and stromal cells this work adds new insights in the mechanism of tumor angiogenesis and of its possible inhibition.

**Supplementary Information:**

The online version contains supplementary material available at 10.1007/s10456-021-09815-4.

## Introduction

Embryonic stem cells (ES) are pluripotent cells derived from the inner cell mass of the growing blastocyst [1]. Due to their intrinsic capability to generate somatic cells of all three germ layers ES cells are the potential source of multiple different cell types to be used for therapeutic purposes [2]. Different procedures have been described to induce stem cell differentiation in vitro exploiting alternative protocols based on the use of chemically defined medium, embryoid bodies (EBs), and co-culture methods. Taking advantage of these multiple choices many efforts were undertaken in these past years to obtain endothelial cells from ES cells for human cell therapy [3]. Indeed, the use of ES cells and of their different protocols of differentiation falls into the rich collection of methodologies used to analyze the angiogenesis process [4]. For instance, in a serum-free chemically defined medium, in presence of extracellular matrices as substrates, endothelial cells were derived from ES cells in response to the exogenous signals FGF2, BMP4, and VEGF added in sequence to the culture medium [5]. The functionality of the ES-derived endothelium was then ascertained in vivo in ischemic mouse model or subcutaneous plugs and grafts [3, 6]. Within the EBs, supported by the addition of VEGF, the differentiating ES cells generate endothelial cells organized in a vessel-like structures thus mimicking the endothelium generation in the embryo [7]. The EBs-derived endothelium was shown to be functional in terms of inflammatory responsiveness, permeability, and sensitivity to shear stress [8, 9]. Another promising approach to obtain endothelium from ES cells relies on the co-culture procedure that allows to recreate a sort of niche that influences the stem cells commitment without the complexity of an EB, thus reaching a good compromise between the use of chemically defined medium and the EBs protocol. The more common stromal cell lines used are OP9 and PA6 cells. OP9 cells are known to direct the differentiation of undifferentiated ES cells toward endothelium [10] and to potentiate the specification of FLK1^+^ endothelial precursors toward mature lymphatic endothelial cells [11]. Even though PA6 stromal cells were identified for their neural inducing activity [12], it has been reported that the presence of BMP4 and all-trans retinoic acid (RA) in the medium of the co-cultures supports the differentiation of ES cells into desmin^+^ mesodermal precursors [13]. According to these observations, we previously described an ES/PA6 co-cultures protocol in which neural and endothelial differentiation can be accomplished simultaneously or alternated with each other depending on the nature of the exogenous signals present in the medium [14]. In a serum-free medium, in absence of any type of signals ES cells originate mainly neurons, whereas the addition of FGF2 allows also the differentiation of endothelium. In presence of BMP4 only endothelial cells arise. On the base of our previous observations, in this work we focused our attention on the endothelium obtained upon FGF2 and BMP4 stimulation by analyzing the patterns of expression of the activated genes.

FGF was the first mesoderm-inducing signal to be identified [15]. Indeed, loss of FGF signaling results in defects in the induction of paraxial mesoderm at gastrula stage [16] and in the lack of brachyury expression with a consequent failure in mesoderm formation [17]. Accordingly, in ES cells studies, FGF signal is important for mesoderm differentiation in both murine [18, 19] and human ES cells [20]. In particular, FGF2 is usually used to differentiate pluripotent human ES cells (hES) into mesoderm in 2D cultures in chemically defined medium where it works in synergy with other cytokines [5]. Precisely, FGF2 drives the primitive streak-like cells obtained by treating hES with GSK-3 inhibitors toward lateral plate mesoderm that can furtherly mature into endothelial cells thanks to the support of other cytokines such as VEGF and BMP4 [3]. BMP4 is another important mesoderm inducer. Using the EBs model Park et al., demonstrated that BMP4 can induce mesoderm and subsequently FLK1^+^ endothelial progenitors but to fully accomplish the differentiation process toward mature endothelium, VEGF is ultimately required [21]. In EBs model BMP4 and WNT3A can activate a localized WNT pathway that in turn polarizes the embryoid body, thus recreating a region enriched of mesoderm that resembles the embryo primitive streak [22]. In line with the observed correlation between BMP4 and mesoderm induction, it has been reported by us [14] and other groups [13] that, when co-cultured with PA6 cells in presence of BMP4, ES cells commitment can be directed toward mesodermal orientation to the expense of ectoderm. Taking a cue from these observations, in this work ES cells were differentiated into endothelium by co-culturing them with PA6 cells in serum-free medium both in presence of FGF2 and in presence of BMP4. Upon each condition, the patterns of genes expression were analyzed and the role played by the identified protagonist molecules and pathways in determining mesoderm commitment was validated through loss of function and rescue experiments.

## Materials and methods

### Cell lines and cultures

The knock in ES cells expressing the enhanced green fluorescent protein under the τ promoter (τ-EGFP) were generously provided by Dr. A.G. Smith [23]. ES cells were cultured as undifferentiated cells onto monolayers of murine embryonic fibroblasts (MEF) growth-arrested with mitomycin C 10 µg/ml (Merck Life Science) in DMEM + GlutaMAX high glucose (Thermofisher Scientific), 15% fetal bovine serum (HyClone), 0.1 mM non-essential aminoacids, 1 mM sodium pyruvate, 10^–4^ M β-mercaptoethanol, penicillin/streptomycin at 50 µg/ml each (Thermofisher Scientific), and 1000 U/ml LIF (Merck Life Science). MEF were purchased from American tissue culture collection (ATCC) (LGC Standards) and cultured according to manufactures, while PA6 stromal cells were provided by the RIKEN BRC through the National Bio-Resource Project of the MEXT, Japan. PA6 cells were cultured in alpha MEM medium supplemented with 2 mM l-glutamine (Euroclone), penicillin/streptomycin at 50 µg/ml each (Thermofisher Scientific, USA), and 10% fetal bovine serum (ATCC) (LGC Standards).

### *2D model of *in vitro* differentiation of ES cells*

PA6 cells were grown in gelatin coated 8-well culture slides (Falcon, Becton Dickinson) until confluence. ES cells were seeded onto PA6 monolayers at a density of 5000 cells/cm^2^ in N2B27 medium [23] composed of Neurobasal medium/DMEM F12 1:1 supplemented with 2 mM glutamine (Euroclone), 50 µg/ml each of penicillin/streptomycin, N2 supplement, B27 supplement, 20 µg/ml insulin (Thermofisher Scientific) 50 µg/ml bovine serum albumin, and 200 µM ascorbic acid (Merck Life Science). When required, 20 ng/ml of human FGF2 and 5 ng/ml of murine BMP4 (R&D Systems) were added at the onset of the culture and withdrawn after 3 days. In some experiments 10 ng/ml of recombinant mouse TGFβ1 (R&D Systems) or 100 ng/ml of recombinant human WNT6 (Abcam) were added to the culture medium and refreshed every two days. When required, 5 µM IWP2 was added to the culture medium and refreshed at every change of medium (Merck Life Science). Medium with fresh insulin was replaced every 2 days.

Loss of function experiments were carried out with shRNA against TGFβ1 (93-sh TRCN0000065993, 97-sh TRCN0000065997) or WNT6 (1807-sh TRCN0000071807, 1804-sh TRCN0000071804, 1803-sh TRCN0000071803, 1805-sh TRCN0000071805, 1806-sh TRCN0000071806) cloned into pLKO.1-puro lentiviral vector (Merck Life Science). Lentiviral particles were prepared as described [24] and used at 20 MOI/cell to infect ES/PA6 co-cultures at the onset of culture. After 3 days, viruses were removed and infected cells were selected with puromycin 1 µg/ml for 24 h (Merck Life Science).

### Indirect immunofluorescence and image analysis

Differentiated ES cells were fixed with phosphate-buffered saline 4% paraformaldehyde (PBS-PFA) (Santa Cruz), treated for 1 h with PBS 0.1% Triton X100 (Merck Life Science) containing 5% donkey serum (Dako North America Inc) and 5% BSA (Merck Life Science) and incubated with primary antibodies for 3 h at 37 °C in a moist chamber. The following primary antibodies were used: anti-βIII-tubulin 1:1000 diluted in PBS (Merck Life Science) and anti-VE-cadherin 1: 100 diluted in PBS (R&D Systems). After rinsing three times with PBS, cells were incubated with 555 AlexaFluor donkey anti-goat and 488 AlexaFluor donkey anti-mouse (Invitrogen, Thermofisher Scientific) at 5 µg/ml for 1 h. After rinsing three times with PBS, nuclei were stained with DAPI and slides were sealed with aqueous mounting solution (Dako North America Inc). Images were captured by using a Leica TCS SPE confocal laser-scanning microscope, analyzed with Leica Confocal Software (LCS; Leica Microsystems). To quantify VE-cadherin^+^ endothelial cells, sequential images, covering the entire surface of a chamber slide well (0.7 cm^2^), were acquired with a Leica AF6000LX workstation. The area occupied by endothelial cells was measured with ImageJ software by using the Angiogenesis Analyzer for ImageJ [25].

### Real-time PCR

Total RNA was extracted with Maxwell RSC miRNA Tissue Kit and processed with Maxwell RSC Instrument (Promega). RNAs were quantified with DeNovix spectrophotometer (DeNovix, Wilmington) and reverse transcribed using High-Capacity cDNA reverse transcription kit (Applied Biosystems, Thermofisher Scientific). cDNAs were used to run a real-time PCR using TaqMan probes specific for the following genes: VE-cadherin, ICAM2, TIE1, TEK, FLK1, TGFβ1, TGFBR1, TGFBR2, ACTA2, CTGF, TBX3, SPP1, ACE, Sema7A, Nanog, BMP4, FGFR3, HES5, T, VEGFA, SNAI1, SNAI2, FGFR2, WNT3, WNT4, WNT6, WNT10A, FZD4, FZD6, LRP5, LRP6, β-catenin, TBP (Applied Biosystems. Thermofisher Scientific). Expression data were analyzed with SDS 2.3 software.

### Gene expression profiling

Total RNA was isolated using the Trizol reagent (Invitrogen, Thermofisher Scientific) and purified using the RNeasy total RNA Isolation Kit (Qiagen). RNA integrity was evaluated by an Agilent 2100 Bioanalyzer (Agilent Technologies). cDNA and biotinylated cRNAs were generated by Illumina Total Prep RNA Amplification Kit (Ambion, Thermofisher Scientific), in accordance to manufacturer’s indications. cRNAs quality and quantification was assessed by Bioanalyzer. Hybridization was carried out on MouseWG-6 v2.0 Expression BeadChip (Illumina Inc.) with 750 ng of biotinylated cRNAs. Array washing, staining, and scanning were performed using standard Illumina protocols. Detection data were processed with the BeadStudio software (Illumina Inc.).

Cubic spline-normalized probe intensity data, together with detection *p*-values, were obtained using GenomeStudio software V2011.01 from Illumina raw data. We selected for subsequent analysis probes with a detection *p*-value < 0.05. For each gene, we retained the associated probe with the largest mean expression value across all samples. For each probe, the log2 signal was converted to the log2 ratio against the global average expression of that probe in all samples. Statistical analyses were performed in the R environment (http://www.r-project.org). In particular, data were clustered using the R pheatmap package and LIMMA (Smyth, 2002) was used to identify the modulated genes. A threshold of | log2 FC |> 1.0 and an adjusted *p*-value < 0.1 were used to select differentially expressed genes. The IPA Ingenuity analysis tool (QIAGEN Inc., https://www.qiagenbioinformatics.com/products/ingenuitypathway-analysis) was used to identify pathways, biological functions, and regulators associated to the modulated genes.

### Statistical analysis

Data are indicated as mean ± SEM. Statistical analysis were performed by using unpaired Students’ *t*-test (two tailed). A *p* < 0.05 was considered significant.

## Results

### Endothelial differentiation of murine ES cells in the ES/PA6 co-culture system

**Fig. 1:**
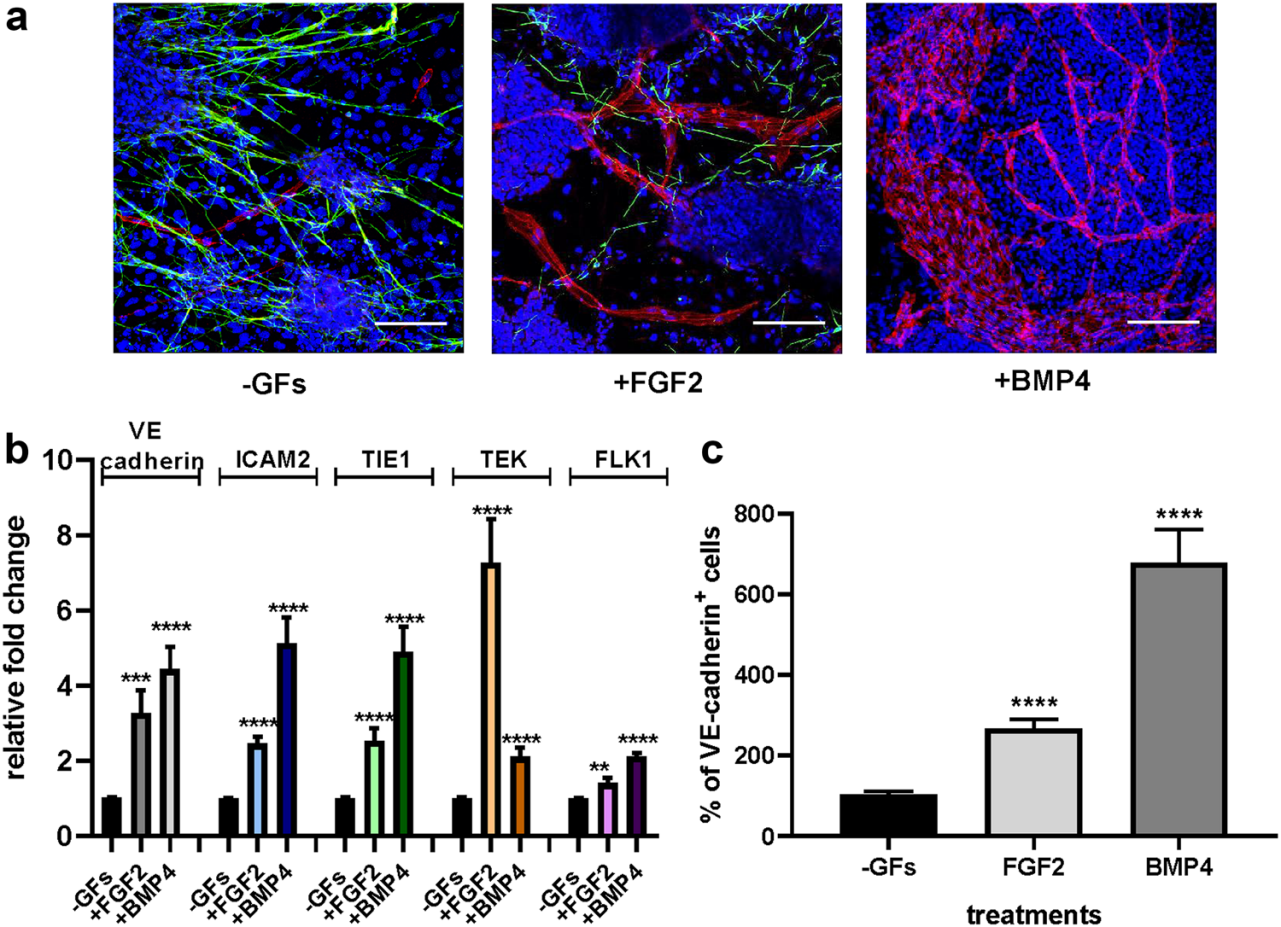
Endothelial differentiation of ES cells occurs in ES/PA6 co-cultures upon FGF2 and BMP4 stimulation. **a** Immunofluorescent stainings of ES/PA6 cell co-cultures performed with anti-VE-cadherin and anti-βIII-tubulin antibodies after 7 days of culture. In absence of growth factors (-GFs) ES cells mainly differentiated into neurons (in green), in presence of FGF2 also mature endothelial cells emerged and formed vessels-like structures (in red), whereas in presence of BMP4 neural differentiation was inhibited in favor of endothelial commitment. Nuclei were evidenced in blue by DAPI staining. Scale bars: 50 µm. **b** Activation of the expression of endothelial markers was evaluated by real-time PCR in response to FGF2 and BMP4 and expressed as relative fold change over the basal level of expression obtained in -GFs medium after normalization to the housekeeping gene TBP (*n* = 10, mean ± SEM; **p < 0.01, ***p < 0.001, ****p < 0.0001). **c** Induction of endothelial differentiation was quantified by measuring the area occupied by VE-cadherin^+^ cells in -GFs medium and in presence of FGF2 and BMP4. The resulting data were normalized to -GFs condition and expressed as percentage of VE-cadherin^+^ cells (*n* = 6, mean ± SEM; ****p < 0.0001)

ES cells were cultured on a monolayer of PA6 cells [12] in serum-free medium in absence of any other type of stimulus (-GFs) or in presence of FGF2 or BMP4. These stimuli were added to the culture medium at the onset of culture thus allowing them to act directly on ES cells themselves or indirectly by activating the stromal component. The immunofluorescence analysis of the co-cultures performed with an anti-VE-cadherin antibody revealed the presence of mature VE-cadherin^+^ endothelial cells, both upon FGF2 and BMP4 stimulation (Fig. 1a). In -GFs medium, despite the presence of few VE-cadherin^+^ cells, cell fate was mainly committed toward neural differentiation, as demonstrated by the presence of βIII-tubulin^+^ cells. The presence of FGF2 was not limitative of neural commitment while BMP4 strictly excluded the maturation of βIII-tubulin^+^ cells, thus favoring mesoderm derivatives, as previously shown [12]. Gene expression analysis by real-time PCR of endothelial markers confirmed the acquisition of endothelial commitment by the differentiating ES cells, being mRNA levels of the endothelial markers VE-cadherin, ICAM2, TIE1, TEK, and FLK1 significantly upregulated in presence of FGF2 and BMP4 respect to -GFs condition (Fig. 1b). To better quantify the inductive effect exerted by the cytokines on the endothelial commitment embraced by the ES cells in the co-cultures, the areas occupied by the VE-cadherin^+^ cells upon all the three different culture conditions were measured and analyzed by image analysis. The immunofluorescence analysis of vascular structures formed in cells challenged with FGF2 and BMP4 compared to what obtained in -GFs medium (Fig. 1c) indicated that BMP4 sustained the endothelial differentiation of the ES cells more efficiently than FGF2, according to its well-known role of mesoderm inducer [26].

### Gene expression analysis of ES/PA6 co-cultures in response to FGF2 and BMP4

In order to unveil the gene pathways involved in FGF2- and BMP4-driven endothelial differentiation, gene expression analysis was performed on ES/PA6 co-cultures and on pure cultures of PA6 cells to better distinguish the autocrine ES cells signals from the paracrine ones coming from the stroma. Moreover, in order to describe the temporal progression of gene expression that sustains the endothelial differentiation process, RNA samples were analyzed after 3 and 7 days of culture, times at which the endothelial differentiation was respectively progressing and fully accomplished. Finally, to obtain an overview of the immediate early genes activated by the stroma in response to the exogenous stimuli, gene expression profiles were generated from cultures of pure PA6 cells after 3 h of stimulation with FGF2 or BMP4. A comparative transcriptome analysis of FGF2- or BMP4-treated cells versus -GFs condition was performed by using LIMMA [27] and the results are summarized in a cartoon (Supplementary Fig. 1a). Within the ES/PA6 co-cultures, FGF2 could alter the expression of 189 genes just after 3 days of culture, while BMP4 required four more days to modulate a significant number of genes. This trend was maintained during time, being FGF2 able to modulate twice as many genes compared to BMP4 after 7 days of culture. PA6 cells alone responded very early to FGF2. After 3 h of incubation, FGF2 promoted the induction of 253 genes and the repression of 138 transcripts, thus indicating that at the onset of culture the differentiating ES cells might receive several paracrine signals from the stroma. On the contrary, BMP4 could elicit an appreciable gene response only after 3 days of culture. A list of the genes modulated by FGF2 and BMP4 in pure cultures of PA6 cells at day 3 of culture is illustrated in Online Resource 1. After 7 days of culture, pure PA6 cells became unresponsive to both FGF2 and BMP4, thus making null their contribution to the co-culture system. After 3 days of culture, upon FGF2 stimulation, PA6 pure cells and ES/PA6 co-cultures share 38 genes while just 4 genes are in common between the two cell types upon BMP4 treatment (Supplementary Fig. 1b). The genes exclusively modulated in the co-cultures were probably involved in the differentiation program. To summarize, this gene expression analysis suggested that during the time window of 7 days of culture FGF2 could elicit an earlier and more robust response than BMP4, being accompanied by an immediate activation of the stroma.

**Fig. 2:**
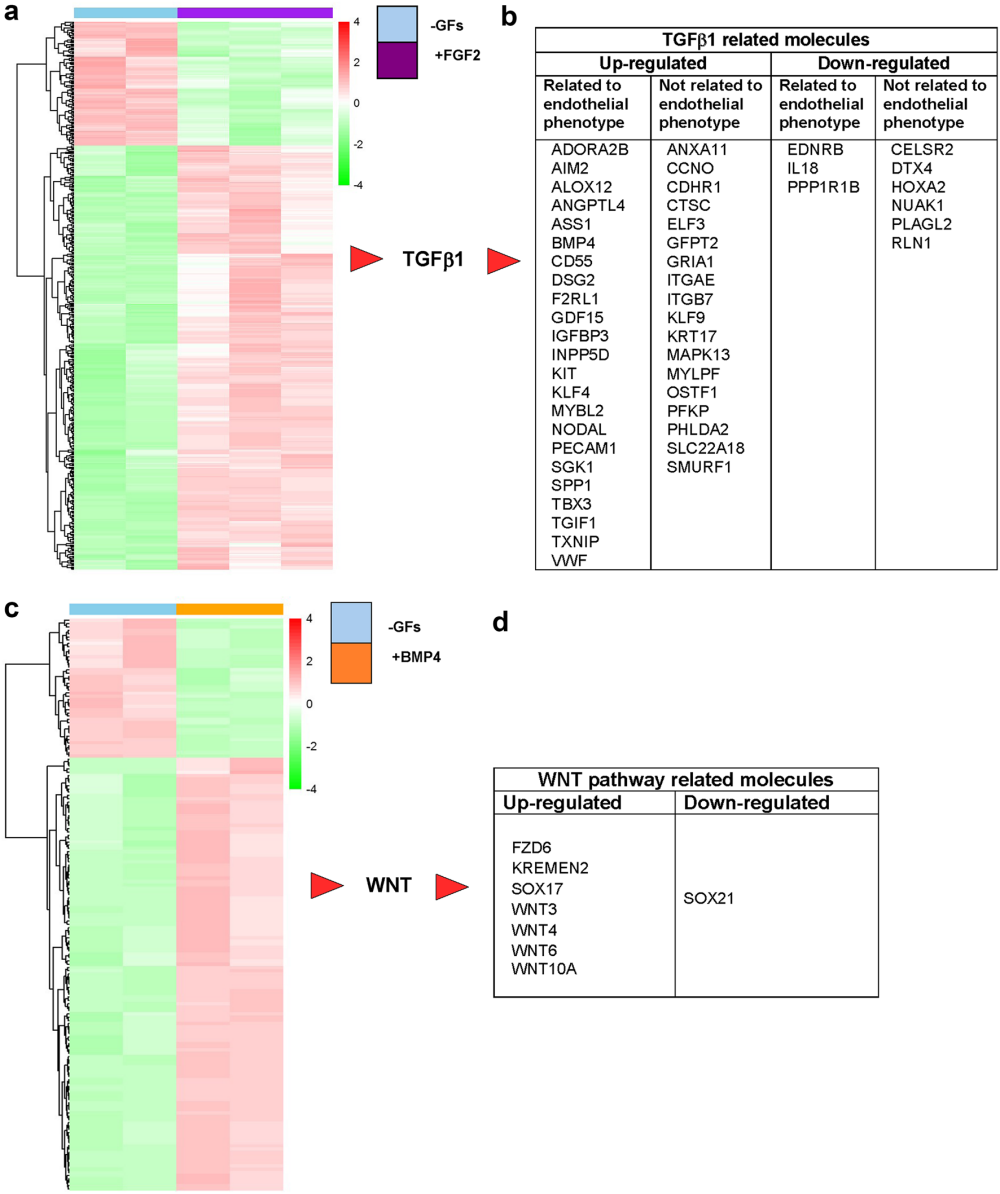
Activation of TGFβ1 pathway by FGF2 and WNT pathway by BMP4 in ES/PA6 co-cultures. **a** Heat map showing clustering of murine differentially expressed genes between FGF2-treated ES/PA6 co-cultures and co-cultures grown in -GFs medium for 7 days. Red: up-regulated genes; green: down-regulated genes. **b** The analysis of the differentially expressed genes by IPA Ingenuity software identified TGFβ1 as the most represented upstream regulator activated in response to FGF2. TGFβ1 indirect interactors were identified among the differentially expressed genes and listed accordingly to their modulation (up-regulated or down-regulated) and subdivided in two groups depending on the consistency with the observed endothelial phenotype. **c** Heat map showing clustering of murine differentially expressed genes between BMP4-treated ES/PA6 co-cultures and co-cultures grown in -GFs medium for 7 days. Red: up-regulated genes; green: down-regulated genes.** d** The analysis of the differentially expressed genes by IPA Ingenuity software identified WNT signaling as the most probable pathway activated by BMP4. The WNTs ligands and WNT-related molecules that were found to be differentially expressed in ES/PA6 co-cultures upon BMP4 stimulation are listed accordingly to their modulation (up-regulated or down-regulated)

The modulation of gene expression after 7 days of treatments is well visualized by the heat map shown in Fig. 2a. The enriched biological functions of up- and down-regulated genes were determined by IPA Ingenuity analysis. Such analysis evidenced the transforming growth factor β1 (TGFβ1) as one of the most important upstream regulators along with its indirect interactors all modulated by FGF2 (Supplementary Fig. 2). Figure 2b shows only those interactors, up-regulated and down-regulated, that relate to TGFβ1 with consistency: they are subdivided in two groups according to their relation or not relation with the observed endothelial phenotype. The importance of TGFβ1 in FGF2-induced endothelial differentiation was evident already from day 3: among the differentially expressed genes shown in the heat map (Supplementary Fig. 4a) TGFβ1 was evidenced as one of the most represented top upstream regulators with its indirect interactors some of which are consistent with the endothelial phenotype (Supplementary Fig. 4b and 4c).

A similar analysis was conducted on BMP4-treated samples after 7 days of culture. Among the 173 modulated genes shown in the heat map (Fig. 2c) IPA Ingenuity analysis identified eight genes belonging to the pathway of WNT (Fig. 2d), thus evidencing WNT pathway as the most representative molecular mechanism induced by BMP4 treatment (Supplementary Fig. 3). To summarize, the bioinformatics analysis of the output data of the array suggested that TGFβ1 might be involved in the endothelial differentiation of the ES cells in presence of FGF2, whereas WNT signaling was indicated as one of the possible protagonist for BMP4-driven endothelial differentiation.

To validate the array data, the levels of some of the messengers randomly chosen among those that were found to be modulated in the array were analyzed by real-time PCR. As shown in Supplementary Fig. 5a the messengers of TGFβ1, TBX3 (T-box transcription factor TBX3), SPP1 (secreted phosphoprotein 1), ACE (angiotensin converting enzyme), Sema7A (semaphorin 7A), and Nanog (homeobox protein Nanog) genes that, according to the array, were modulated after 3 days of co-culture upon FGF2 stimulation, were confirmed to be increased also by real-time PCR. Similarly, in accord with microarray results, after 7 days of cultures FGF2 induced the up-regulation of BMP4, the tyrosine kinase TEK, TBX3, SPP1, and TGFβ1 genes (Supplementary Fig. 5b) and the down-regulation of FGFR3 and of the transcription factor HES5 (Supplementary Fig. 5c).

### TGFβ1* in FGF2-induced endothelial differentiation*

**Fig. 3:**
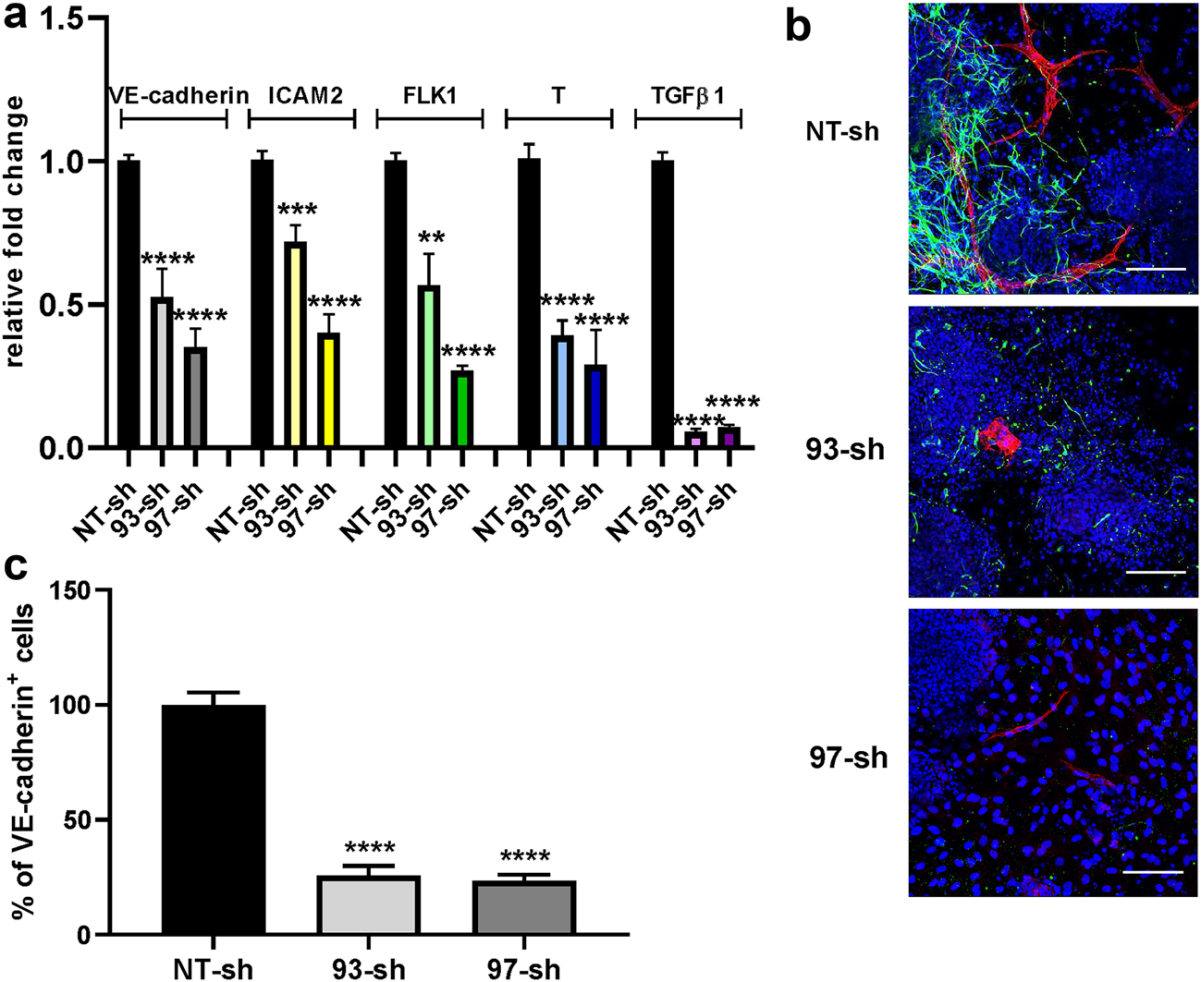
Inhibition of endothelial differentiation by silencing TGFβ1 in ES/PA6 co-cultures upon FGF2 stimulation. TGFβ1 expression was knocked out by adding lentiviral vectors carrying TGFβ1specific shRNAs (93-sh, 97-sh) or a non-transducing shRNA (NT-sh) to the culture medium of ES/PA6 co-cultures. **a** After 7 days, the modulation of the expression of endothelial markers and of TGFβ1gene was evaluated by real-time PCR in response to FGF2 and expressed as relative fold change over the levels of expression obtained from ES/PA6 co-cultures transduced with NT-sh. Data were normalized to the housekeeping gene TBP. All gene examined were significantly down-regulated by silencing TGFβ1 (*n* = 4, mean ± SEM; **p < 0.01, ***p < 0.001, ****p < 0.0001). **b** Immunofluorescent stainings of ES/PA6 cell co-cultures transduced with NT-sh, 93-sh, and 97-sh and grown in presence of FGF2 were performed with anti-VE-cadherin and anti-βIII-tubulin antibodies to detect endothelial cells (in red) and neurons (in green). Nuclei were evidenced in blue by DAPI staining. Scale bars: 50 µm. **c** The inhibition of the endothelial differentiation in TGFβ1-silenced co-cultures was quantified by measuring the area occupied by VE-cadherin^+^ cells. The resulting data were normalized to those obtained by NT-sh transduced co-cultures and expressed as percentage of VE-cadherin^+^ cells (*n* = 6, mean ± SEM; ****p < 0.0001)

**Fig. 4:**
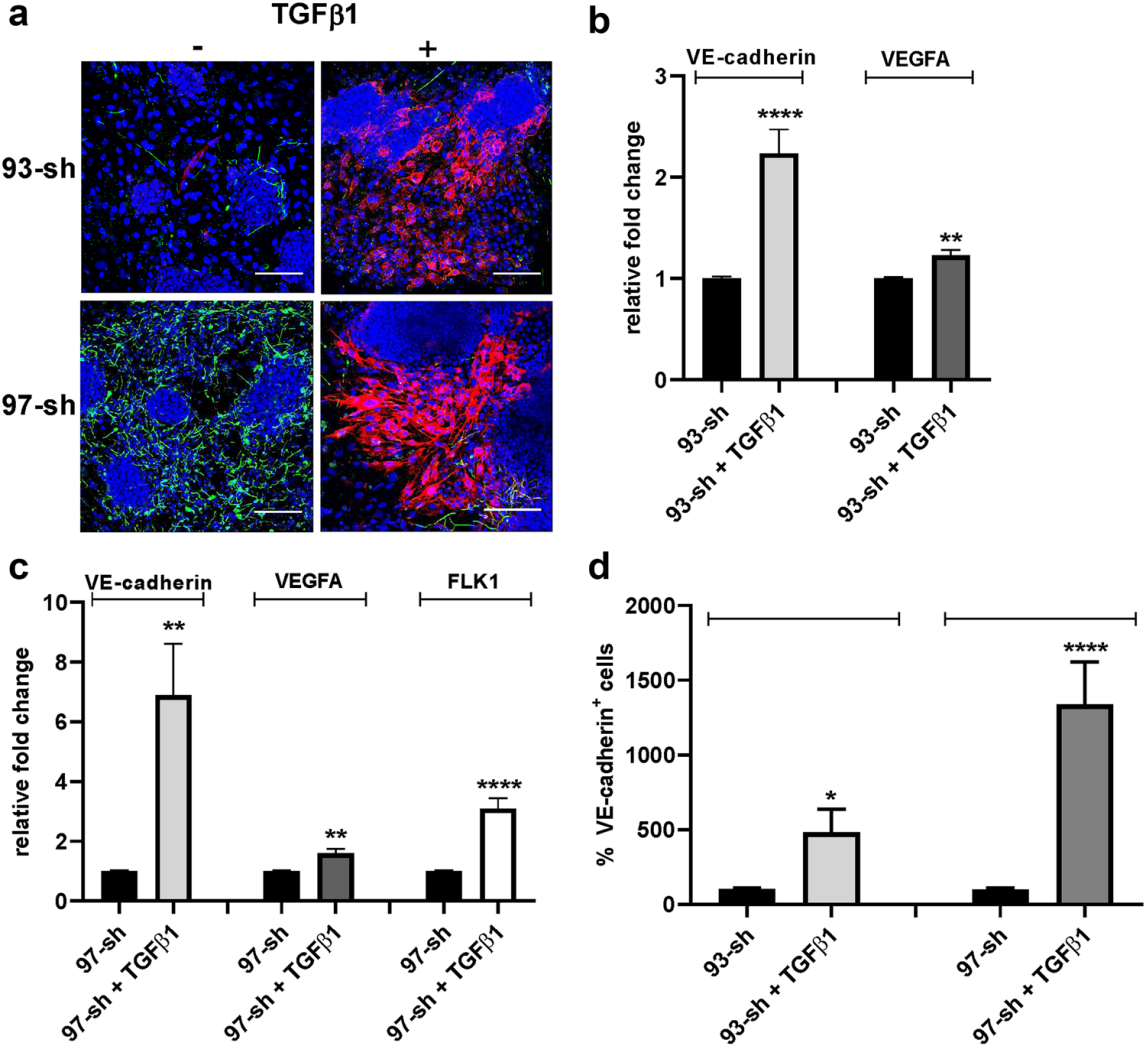
Exogenous recombinant TGFβ1 can rescue the inhibition of FGF2-driven endothelial differentiation induced by silencing TGFβ1in ES/PA6 co-cultures. **a** Immunofluorescent stainings of ES/PA6 co-cultures treated with FGF2 and transduced with the two TGFβ1 specific shRNAs 93-sh and 97-sh were performed with anti-VE-cadherin and anti-βIII-tubulin antibodies, in absence or in presence of recombinant TGFβ1. The addition of recombinant TGFβ1 to the culture medium resumed the differentiation of ES cells into VE-cadherin^+^ cells (in red). Nuclei were evidenced in blue by DAPI staining. Scale bars: 50 µm. After 7 days of culture, the modulation of endothelial markers expression in ES/PA6 co-cultures transduced with 93-sh (**b**) or 97-sh (**c**), was evaluated by real-time PCR in response to recombinant TGFβ1 and expressed as relative fold change over the level of expression obtained in untreated ES/PA6 co-cultures. Data were normalized to the housekeeping gene TBP (*n* = 3, mean ± SEM; **p < 0.01, ****p < 0.0001), **d** The capacity of exogenous TGFβ1 to resume the endothelial commitment of TGFβ1-silenced ES/PA6 co-cultures was quantified by measuring the area occupied by VE-cadherin^+^ cells. The resulting data were normalized to those obtained in absence of recombinant TGFβ1 and expressed as percentage of VE-cadherin^+^ cells (*n* = 5, mean ± SEM; *p < 0.05, ****p < 0.0001)

The role played by TGFβ1 in FGF2-induced endothelial differentiation was evaluated by analyzing the differentiation process and the final phenotype acquired by ES/PA6 co-cultures in its absence. To this purpose, TGFβ1 expression was silenced contemporary in both PA6 and ES cells by adding at the onset of culture a lentiviral vector carrying the correspondent specific shRNA. Being aware of possible off-target effects, two different shRNA sequences were tested. TGFβ1 gene knockdown efficiency, as well as the levels of expression of endothelial markers VE-cadherin, ICAM2, FLK1, and T (brachyury) were evaluated by real-time PCR after 7 days of co-culturing ES cells in the presence of FGF2. Besides targeting TGFβ1 gene expression, both shRNA sequences 93-sh and 97-sh caused the down-regulation of the expression of endothelial markers suggesting an inhibition of endothelial differentiation (Fig. 3a). Indeed, the immunofluorescence analysis of ES/PA6 co-cultures silenced for TGFβ1 expression and treated with FGF2 revealed the almost total absence of VE-cadherin^+^ cells after 7 days of culture (Fig. 3b). The quantification of the areas occupied by the VE-cadherin^+^ cells confirmed a 70–80% reduction of the differentiated endothelium thus reinforcing the relevance of TGFβ1 in determining the endothelial fate (Fig. 3c). The specificity of the involvement of TGFβ1 in FGF2-induced endothelial differentiation was further investigated by evaluating the capacity of exogenous recombinant TGFβ1 to recover the endothelial phenotype in TGFβ1-silenced ES/PA6 co-cultures upon FGF2 stimulation. As shown in Fig. 4a, in the presence of exogenous TGFβ1 ES cells recovered the capacity to maturate into VE-cadherin^+^ cells that mostly appeared as disorganized cell clusters with few vessels-like structures. The loss of organization of the recovered VE-cadherin^+^ endothelial cells is in line with what previously observed by Goumans et al., in engineered EBs overexpressing the wild type form of TGFBR2 [28]. The rescue of the endothelial phenotype by exogenous TGFβ1 in TGFβ1-silenced ES/PA6 co-cultures was confirmed by the raise of VE-cadherin and VEGFA transcripts respect to the ones measured in co-cultures silenced with 93-sh (Fig. 4b) and by the raise of VE-cadherin, VEGFA and FLK1 transcripts when recombinant TGFβ1 was added to co-cultures silenced with 97-sh (Fig. 4c). Furthermore, the quantification by image analysis of the areas occupied by VE-cadherin^+^ cells proved the reacquisition of the endothelial commitment of the differentiating ES cells in the presence of exogenous TGFβ1 (Fig. 4d). In line with the involvement of TGFβ1 in FGF2-driven endothelial differentiation, TGFBR1 and TGFBR2, other than TGFβ1 itself, were found to be expressed and up-regulated by FGF2 in both ES/PA6 co-cultures and pure cultures of PA6 cells along with the TGFβ1 target genes SNAI1 and CTGF (Supplementary Fig. 6 and 7). These data demonstrate that in ES/PA6 co-culture system FGF2 elicits endothelial commitment in the differentiating ES cells through TGFβ1 action.

### *Role of the stroma-derived *TGFβ1* in endothelial differentiation*

**Fig. 5:**
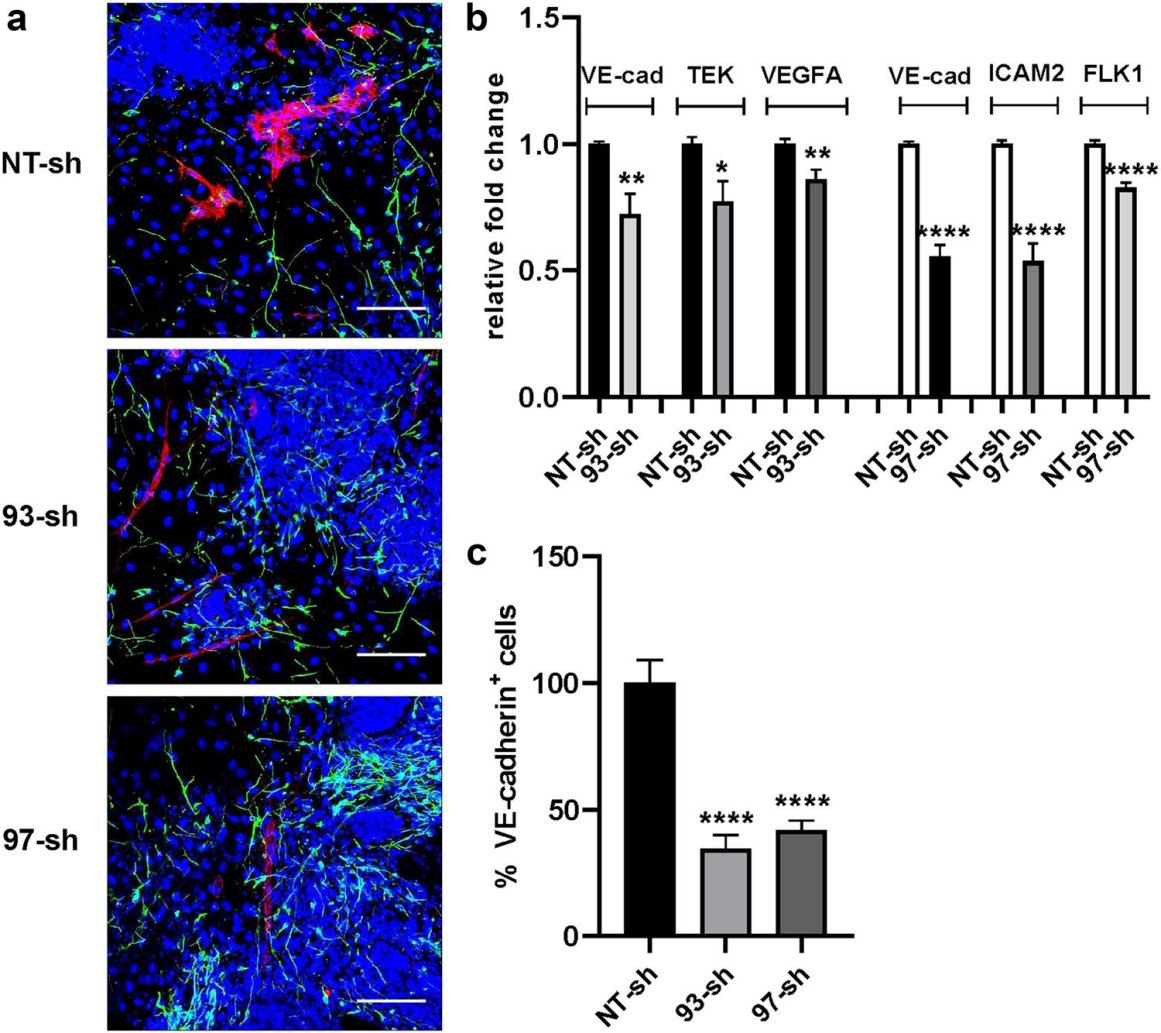
Role of paracrine TGFβ1 released by stromal cells in FGF2-driven endothelial differentiation in ES/PA6 co-cultures. ES cells were co-cultured upon FGF2 stimulation with PA6 stromal cells that were previously transduced with NT-sh or with the two TGFβ1 specific shRNAs 93-sh and 97-sh. **a** Immunofluorescent stainings of co-cultures with anti-VE-cadherin and anti-βIII-tubulin antibodies evidenced a decrease in the maturation of VE-cadherin^+^ cells (in red). Nuclei were evidenced in blue by DAPI staining. Scale bars: 50 µm. **b** After 7 days of culture, upon FGF2 stimulation, the modulation of the expression of endothelial markers was evaluated by real-time PCR in co-cultures settled with PA6 cells silenced for TGFβ1 expression. Data were expressed as relative fold change over the levels of expression obtained by using NT-sh-transduced PA6 cells as feeders. Data were normalized to the housekeeping gene TBP (*n* = 3, mean ± SEM; *p < 0.05, **p < 0.01, ****p < 0.0001). **c** The decrease of endothelial differentiation caused by the lack of stromal TGFβ1 was quantified by measuring the area occupied by VE-cadherin^+^ cells. The resulting data were normalized to those obtained by using PA6 cells transduced with NT-sh and expressed as percentage of VE-cadherin^+^ cells (*n* = 3, mean ± SEM; ****p < 0.0001)

Being the stromal component, PA6 cells sent important signals to the ES cells that were differentiating above them. Indeed, pure cultures of PA6 cells treated with FGF2 actively responded with a significant modification of transcriptome starting from 3 h of treatment (Supplementary Fig. 8a). Out of the 391 modulated genes, the levels of expression of some of them, such as VEGFA, Sema7A, SNAI1, SNAI2, FGFR2, and TGFβ1 were confirmed by real-time PCR (Supplementary Fig. 9a). Among all these genes, TGFβ1 was evidenced as one of the most represented upstream regulator, as detected by IPA Ingenuity analysis (Supplementary Fig. 8b). Similar results were obtained also with pure cultures of PA6 cells after 3 days of treatment with FGF2 (Supplementary Fig. 8c, 8d, and 9b). On this base, it was important to distinguish between the contribution given to the endothelial commitment by paracrine TGFβ1 derived from the stroma, from the autocrine signal coming from the ES cells themselves [29]. To this purpose, ES cells were co-cultured in presence of FGF2 with PA6 cells in which the expression of TGFβ1 was previously silenced with TGFβ1-shRNAs (Supplementary Fig. 10). After 7 days of culture in presence of FGF2, the VE-cadherin^+^ cells that differentiated on TGFβ1-silenced PA6 cells were limited to few small branches, as visualized by immunofluorescence (Fig. 5a). A real-time PCR analysis performed on total RNAs purified from ES/PA6 co-cultures prepared with PA6 cells transduced with TGFβ1-shRNAs or with NT-shRNA confirmed a down-regulation of the messengers of endothelial genes, such as TEK, VEGFA, VE-cadherin, ICAM2, FLK1 (Fig. 5b). The inhibitory effect pursued by silencing the paracrine TGFβ1 was better quantified by measuring the areas occupied by VE-cadherin^+^ cells in all co-cultures and by comparing the values obtained with TGFβ1-silenced PA6 cells (93-sh and 97-sh) with the ones of control PA6 cells (NT-sh) (Fig. 5c). These data show that the blockade of TGFβ1 paracrine signal counteracts the potential of ES cells endothelial differentiation of about 50%, thus underling the importance of the stroma within the co-cultures.

### Involvement of WNT pathway in BMP4-induced endothelial differentiation

**Fig. 6:**
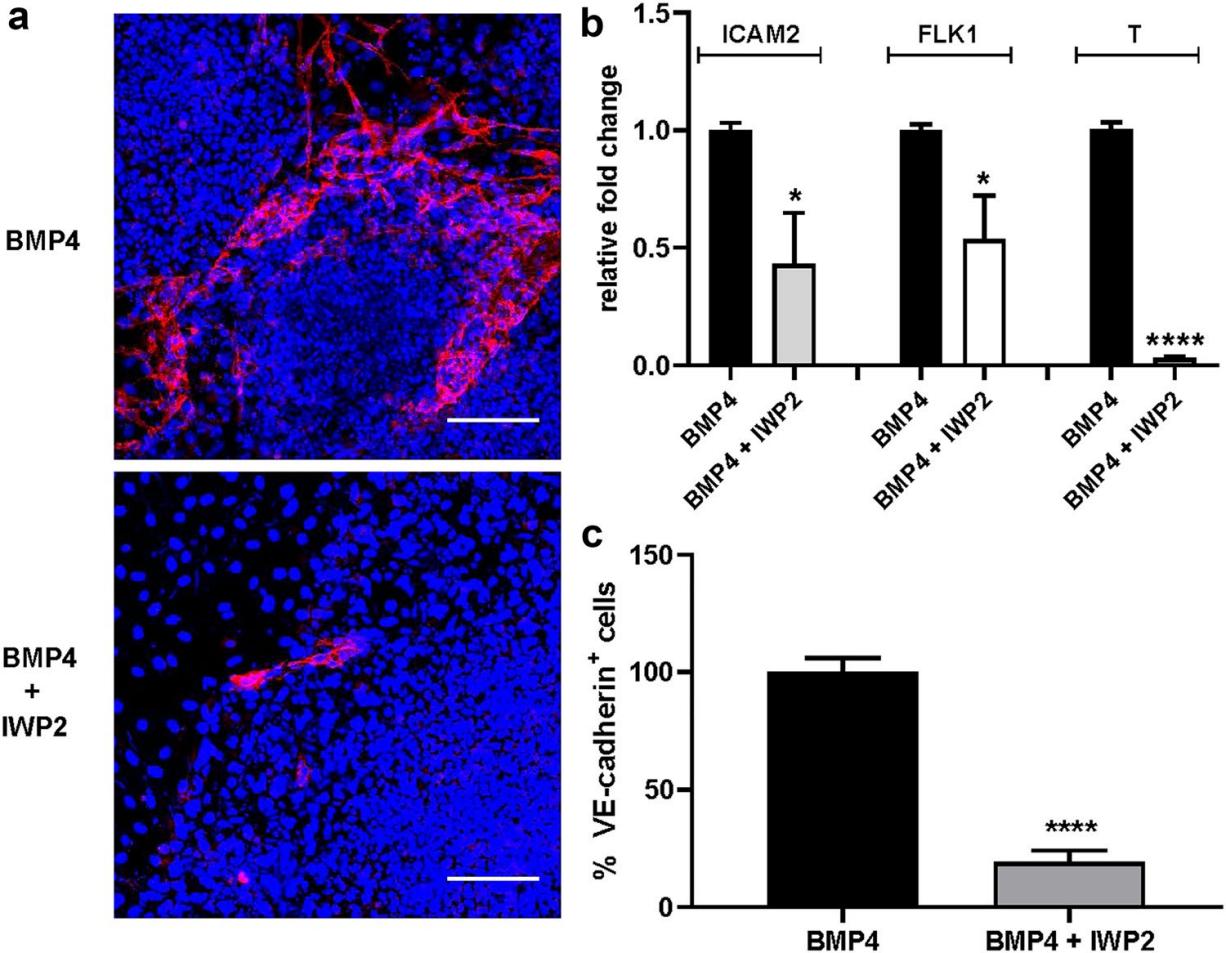
Inhibition of WNT signaling causes a decreased efficiency of the endothelial differentiation induced by BMP4. **a** ES/PA6 co-cultures were cultured for 7 days upon BMP4 stimulation in presence or in absence of the chemical WNT inhibitor IWP2. Immunofluorescent stainings performed with anti-VE-cadherin antibody evidenced how IWP2 affected the endothelial differentiation driven by BMP4, being the VE-cadherin^+^ cells (in red) significantly reduced in IWP2-treated co-cultures respect to the untreated ones. Nuclei were evidenced in blue by DAPI staining. Scale bars: 50 µm. **b** The modulation of the expression of endothelial markers was evaluated by real-time PCR in ES/PA6 co-cultures stimulated with BMP4 and treated with IWP2. The data were normalized to the housekeeping gene TBP and expressed as relative fold change over the levels of expression detected in untreated co-cultures (*n* = 2, mean ± SEM; *p < 0.05, ****p < 0.0001). **c** Quantification of the inhibition of BMP4-driven endothelial differentiation by IWP2 was performed by measuring the area occupied by VE-cadherin^+^ cells. The data were normalized against those obtained with untreated co-cultures and expressed as percentage of VE-cadherin^+^ cells (*n* = 3, mean ± SEM; ****p < 0.0001)

**Fig. 7:**
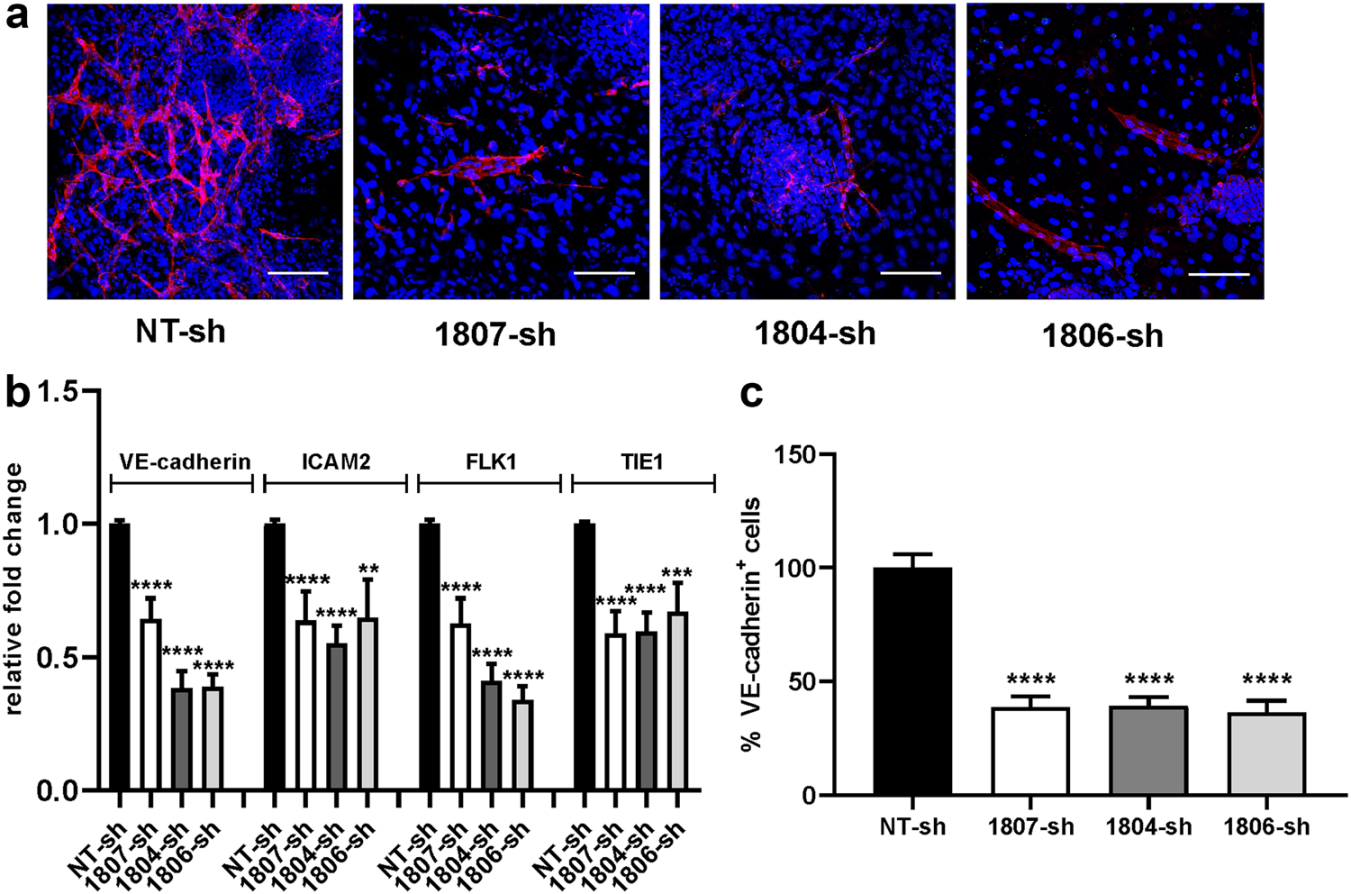
Inhibition of endothelial differentiation by silencing WNT6 in ES/PA6 co-cultures upon BMP4 stimulation. WNT6 expression was silenced in ES/PA6 co-cultures by adding to the culture medium lentiviral particles containing lentiviruses carrying WNT6-specific shRNAs (1807-sh, 1806-sh, 1804-sh), whereas a non-transducing shRNA (NT-sh) was used as control. **a** After 7 days of culture and BMP4 stimulation, a significant decrease of endothelial cells maturation was evident in WNT6-silenced ES/PA6 co-cultures compared to the control (NT-sh), as evidenced by immunofluorescent staining obtained with the anti-VE-cadherin antibody (in red). Nuclei were evidenced in blue by DAPI staining. Scale bars: 50 µm. **b** The levels of expression of endothelial markers were evaluated by real-time PCR in ES/PA6 co-cultures stimulated with BMP4 and silenced for WNT6 expression. After normalization to the housekeeping gene TBP, data were expressed as relative fold change of co-cultures transduced with the specific WNT6 shRNAs 1807-sh, 1806-sh, and 1804-sh over the control co-cultures transduced with NT-sh (*n* = 3, mean ± SEM; **p < 0.01, ***p < 0.001, ****p < 0.0001). **c** The inhibition of endothelial differentiation caused by silencing WNT6 expression was quantified by measuring the area occupied by VE-cadherin^+^ cells. The data derived from ES/PA6 co-cultures transduced with each of the three WNT6-specific shRNAs 1807-sh, 1806-sh, and 1804-sh were normalized against those obtained with control co-cultures transduced with NT-sh (*n* = 3, mean ± SEM; ****p < 0.0001)

Similar to FGF2, exogenous BMP4 can induce endothelial differentiation of the ES cells within the co-cultures with the difference that it is characterized by a richer production of mature VE-cadherin^+^ endothelium to the expense of neural differentiation. These findings agree with the specific activities of BMP4, a well-known mesoderm inducer and neural inhibitor [6, 23]. The involvement of WNT pathway in BMP4-induced endothelial differentiation suggested by bioinformatics analysis of transcriptome data (Fig. 2c, d), was confirmed by real-time PCR performed on total RNA purified from ES/PA6 co-cultures after 7 days of treatment with BMP4 or after 7 days of culture in -GFs medium. In agreement with gene expression results, we confirmed that four members of WNT family were up-regulated (WNT3, WNT4, WNT6, and WNT10A) along with FZD6, which is one of the receptors of WNT ligands (Supplementary Fig. 11a). Few other genes, randomly chosen among all the genes that, according to the array, were modulated, were confirmed to be activated, such as SNAI2, TBX3, SPP1, TEK while HES5 was down-regulated (Supplementary Fig. 11b). The role played by WNT pathway in BMP4-induced endothelial differentiation was evaluated by analyzing the impact that a blockade of WNT signaling might have on the differentiation process of the ES cells. To this purpose ES/PA6 co-cultures were grown for 7 days in presence of BMP4 and upon the action of the chemical WNT inhibitor IWP2 [30]. By blocking WNT palmitoylation through the inactivation of porcupine, a member of the membrane-bound O-acyltransferases [31], IWP2 inhibits WNT activation and secretion. The immunofluorescent analysis performed with an anti-VE-cadherin antibody of ES/PA6 cells co-cultures upon BMP4 stimulation revealed that the presence of IWP2 severely impaired the maturation of VE-cadherin^+^ cells (Fig. 6a). The analysis by real-time PCR of the mRNA levels of the endothelial markers ICAM2, FLK1, and T confirmed the inhibitory effect of IWP2 (Fig. 6b). Indeed, the quantification of the VE-cadherin^+^ cells showed a significant reduction of the areas occupied by the endothelial cells in presence of IWP2 compared to the endothelium developed in its absence (Fig. 6c). On the contrary the presence of IWP2 did not affect the FGF2-induced endothelial differentiation (Supplementary Fig. 12) thus confirming that, differently from BMP4, FGF2 did not require WNT signaling to address endothelial fate in ES cells. Being aware of possible generic effects of a powerful molecule such as a chemical inhibitor, the contribution of WNT activity to BMP4-induced endothelial differentiation was better defined by specifically inhibiting a single WNT molecule. The fact that, among the four WNT modulated genes, WNT 6 was activated up to almost two hundred times, made it a favorite candidate for a focused inhibitory strategy. Indeed WNT6 expression was gradually but consistently up-regulated by BMP4 in ES/PA6 co-cultures during time along with the WNT receptors FZD4 and LRP6 (Supplementary Fig. 13). On the contrary pure cultures of PA6 cells did not express WNT6 (data not shown) and WNT receptors were not significantly modulated by BMP4 (Supplementary Fig. 14). On this base, WNT6 expression was silenced at the onset of co-cultures by specific shRNAs. Being aware of possible off-target effects, five different shRNA sequences were assayed. WNT6 knockdown efficiency was evaluated by real-time PCR after 7 days of culturing ES/PA6 cells co-cultures in the presence of BMP4. The levels of WNT6 gene expression were expressed as fold change of mRNA levels over the one obtained with control cells transduced with NT-sh (Supplementary Fig. 15). Out of the five shRNAs sequences, the less efficient 1803-sh and 1805-sh were not considered in the experiments that followed. The immunofluorescence analysis performed on ES/PA6 cells co-cultures stimulated with BMP4 and after 7 days of culture revealed a significant reduction of the VE-cadherin^+^ cells in response to WNT6 silencing respect to control co-cultures transduced with NT-sh (Fig. 7a). In accord to this trend, as a consequence of WNT6 blockade, the expression of messengers of the endothelial markers VE-cadherin, ICAM2, FLK1, and TIE1 was significantly downregulated respect to the basal levels obtained with control co-cultures (Fig. 7b). Accordingly, in WNT6-silenced co-cultures the areas occupied by VE-cadherin^+^ cells were significantly reduced (50%) compared to the endothelium developed in control co-cultures (Fig. 7c). The fact that the reduction of endothelium was not absolute suggested that WNT6 was not the only WNT molecule involved, especially since WNT10A was confirmed to be up-regulated of a hundred times in response to BMP4 (Supplementary Fig. 11a).

**Fig. 8:**
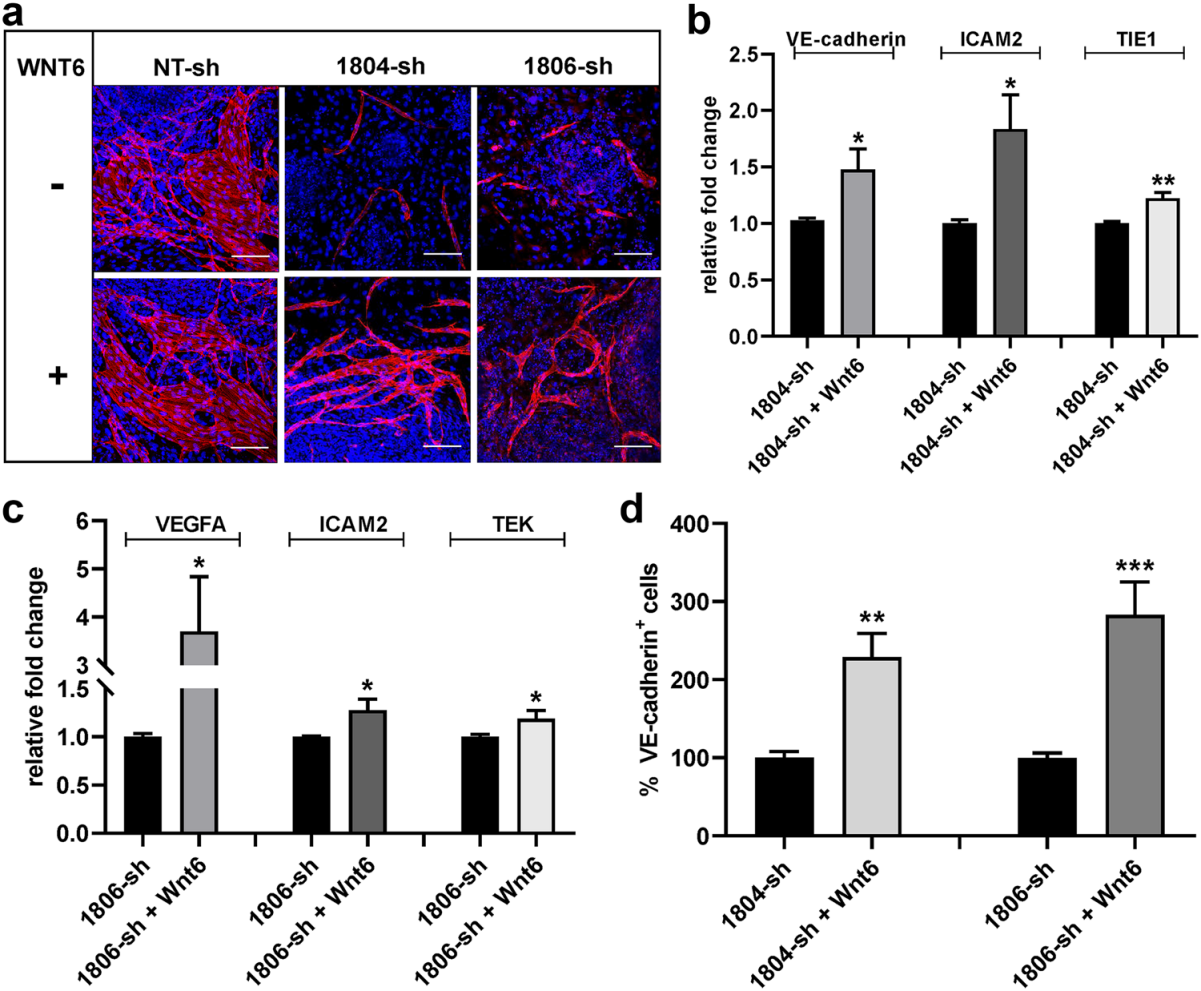
Exogenous recombinant WNT6 can rescue the inhibition of BMP4-driven endothelial differentiation induced by silencing WNT6 in ES/PA6 co-cultures. **a** Immunofluorescent stainings of ES/PA6 co-cultures transduced with the two WNT6-specific shRNAs 1804-sh and 1806-sh or with non-transducing NT-sh in presence or in absence of exogenous recombinant WNT6. The anti-VE-cadherin antibody evidenced the capacity of recombinant WNT6 to resume the efficiency of the endothelial differentiation that was partially impaired by the loss of endogenous WNT6. Endothelial cells were evidenced in red while nuclei in blue by DAPI staining. Scale bars: 50 µm. As evidenced by real-time PCR, exogenous WNT6 could resume the expression of endothelial genes in ES/PA6 co-cultures in which the endogenous WNT6 was silenced with 1804-sh (**b**) or with 1806-sh (**c**). After normalization to the housekeeping gene TBP, data were expressed as relative fold change of co-cultures treated with WNT6 over the untreated ones (*n* = 3, mean ± SEM; *p < 0.05, **p < 0.01 and *n* = 2, mean ± SEM; *p < 0.05). **d** The capacity of exogenous WNT6 to resume the endothelial commitment of WNT6-silenced ES/PA6 co-cultures was quantified by measuring the area occupied by VE-cadherin^+^ cells. The resulting data were normalized to those obtained in absence of recombinant WNT6 and expressed as percentage of VE-cadherin^+^ cells (*n* = 3, mean ± SEM; **p < 0.01, ***p < 0.001)

The specific contribution given by WNT6 to the BMP4-driven endothelial commitment of ES cells was better investigated by evaluating the capacity of exogenous recombinant WNT6 to recover the impaired endothelial phenotype observed upon WNT6 gene silencing. To this purpose, recombinant WNT6 was added, at the onset of culture, to the culture medium of ES/PA6 co-cultures in which WNT6 expression was inhibited by specific WNT6 shRNAs and the accomplishment of the endothelial phenotype was evaluated by immunofluorescence analysis. As shown in Fig. 8a, recombinant WNT6 could recover the partial loss of ES cells endothelial commitment caused by silencing the endogenous WNT6. Such phenotype recovery was also mirrored at the gene expression level, since in the presence of exogenous WNT6, the messengers of the endothelial markers VE-cadherin, ICAM2, TIE1, VEGFA, and TEK regained an increased level of expression respect to WNT6-silenced co-cultures grown in absence of WNT6 (Fig. 8b, c). The capacity to rescue the endothelial phenotype by recombinant WNT6 was quantified by measuring the areas occupied by VE-cadherin^+^ cells that developed in ES/PA6 co-cultures after having been silenced for WNT6 expression and treated or not with exogenous WNT6 (Fig. 8d). These data support the role played by WNT6 in BMP4-induced endothelial differentiation.

## Discussion

In this study we differentiated murine ES cells into endothelium by co-culturing them with the stromal PA6 cells in the presence of FGF2 or BMP4 in serum-free medium, as previously described [14], with the purpose to investigate the molecular mechanisms involved in endothelial fate commitment. To accomplish this task, the pattern of genes expression activated by FGF2 and BMP4 in ES/PA6 co-cultures was analyzed by microarray technique followed by comparative transcriptome analysis by LIMMA [27]. Thanks to this approach, we identified TGFβ1 and WNT signaling as respectively relevant cues of FGF2 and BMP4-driven endothelial differentiation (Fig. 2). The involvement of each of these two pathways was confirmed by loss of function experiments that evidenced how endothelial cell fate determination was severely hampered by the absence of TGFβ1 (Fig. 3) and by the blockade of WNT signaling (Fig. 6) in the case of FGF2 and BMP4 stimulation, respectively. More precisely, in the latter case, WNT6 was identified as one of the WNT molecules involved since its down-regulation by lentiviral shRNA significantly affected the endothelial commitment (Fig. 7).

The importance of TGFβ1 for vasculogenesis has been known for some time since Goumans et al., showed that ES cells engineered to express a deleted form of TGFBR2 failed to differentiate into CD31^+^ endothelial cells in EBs [28]. These authors also demonstrated that overexpression of endogenous TGFβ1 in murine EBs resulted in a dysfunctional organization of the endothelial cells that remained compacted into dense cluster in the EBs instead of spreading into vessels-like structures. Similarly, in our rescue experiments, the addition of recombinant TGFβ1 to the TGFβ1-silenced ES/PA6 co-cultures caused the retrieval of VE-cadherin^+^ cells differentiation and the rescue of the phenotype but endothelial cells were not aligned in vessels as well as in the control co-cultures (Fig. 4). The inhibitory effect of TGFβ1 in vessels organization was further confirmed in 3D cultures of EBs included in collagen matrix: the sprouting process of new vessels in response to angiogenic factors could be blocked by recombinant TGFβ1 [32]. These observations confirmed an inducing effect of vasculogenesis by TGFβ1 and an inhibitory role in sprouting angiogenesis, contrary to what observed in human EBs in which TGFβ1 was shown to inhibit endothelial differentiation [33]. In our work, to silence the expression of TGFβ1, the addition of the lentiviral particles carrying the TGFβ1 specific shRNAs was done at the onset of culture, thus targeting both ES cells and PA6 feeders. Since TGFβ1 is produced by PA6 feeders just after three hours of FGF2 stimulation (Supplementary Fig. 6) we wondered if TGFβ1 signal responsible for FGF2-induced endothelial differentiation of the ES cells was an autocrine signal intrinsic of ES cells themselves or a paracrine signal coming from the stroma. Indeed, we demonstrated that TGFβ1-silenced PA6 cells could not support FGF2-driven endothelial differentiation of ES cells as efficiently as wild type PA6 cells (Fig. 5), thus suggesting the importance of a paracrine TGFβ1 signal. On the other hand, the fact that the loss of maturation of VE-cadherin^+^ cells in response to FGF2 of ES cells cultured on TGFβ1-silenced PA6 cells was partial would indicate that a TGFβ1-mediated autocrine loop is important as well. The involvement of TGFβ1 in FGF2-driven endothelial differentiation might be related to a vasculogenesis process, as previously shown in EBs model [32]. In support to this hypothesis we found that, other than TGFβ1, also the expression of SNAI1 gene was upregulated in response to FGF2 (Supplementary Fig. 6) thus indicating that the phenomenon of epithelial mesenchymal transition (EMT) was occurring. EMT is known to be an early and essential step in lineage specification of ES cells because it moves the differentiating cells toward the germ layers specification by promoting the transition from epiblast state to mesenchymal cell phenotype [34]. On these bases in our model FGF2 would activate TGFβ1 that in turn supports the rising mesoderm by promoting EMT of the epiblast cell types within the differentiating ES cells.

WNT signaling plays a pivotal role in the development of the vascular system being essential for the establishment of the primitive streak, the EMT of epiblast cells within the primitive streak and the formation of mesoderm [35, 36]. Indeed, in vivo, the formation of mesoderm is dependent on WNT/β-catenin signaling. Mice lacking the WNT key molecule β-catenin lack mesoderm [37] and WNT3A deletion is associated with a decrease of mesoderm markers expression [38]. Furthermore, it was demonstrated that inhibition of β-catenin expression in the endothelium leads to the death of embryos in utero due to alterations in vascular morphogenesis [39]. Specifically, in the developing brain WNT signaling localizes in the forming blood–brain barrier (BBB) and the conditional loss of β-catenin in endothelial cells after birth causes alterations of vessels permeability confirming that WNT signaling through β-catenin is required for BBB maturation and function [40]. In vitro, in EBs, endogenous WNT signals mediate the formation of mesodermal precursors expressing brachyury and FLK1 in a domain of the EB with primitive streak characteristics: in this domain cells undergo EMT and acquire mesodermal character [22]. The same authors also demonstrated that in serum-free medium the rising of mesoderm was supported by BMP4 through the activation of endogenous WNT signal.

Similarly, in our co-culture system we found that exogenous BMP4 upregulated the expression of endothelial markers, including FLK1, and supported the differentiation of ES cells into VE-cadherin^+^ cells (Fig. 1). We also identified WNT pathway as the most represented pathway induced by BMP4 with four WNT ligands being upregulated among which WNT6 was evidenced as the most activated (Supplementary Fig. 11). In agreement with our findings, WNT pathway was found to be active in FLK1^+^ endothelial precursors purified from EBs and responsible for the formation of vascular network during ES cells differentiation. Differently from our results, in this model the WNT ligands more represented were WNT2 and WNT5A [41]. The presence of WNT ligands in endothelial cells was well documented in vivo and in vitro, covering many of them, from WNT2 to WNT11[42–44] but this is the first work in which a direct involvement of WNT6 is described. Since the specific silencing of WNT6 did not completely block the endothelial maturation of ES cells, the possibility that some of the other WNTs ligands that were up-regulated by BMP4 might be involved must be considered in future works. Other than WNT ligands, we also found up-regulated genes belonging to WNT pathway such as FZD6, FZD4, and LRP6 (Supplementary Fig. 13) thus testifying the activation of WNT signaling by BMP4. We also found the genes of EMT SNAI1 and SNAI2 up-regulated by BMP4 (data not shown) in accord to what previously observed in the primitive streak-like domain of EBs [22]. All these evidences are in sustain of the presence and activation of WNT pathway in BMP4-induced endothelial differentiation of ES cells in our co-culture system.

The ES/PA6 co-culture system represents a versatile and relatively simple in vitro model useful to study the molecular interactions occurring between the environment and the differentiating stem cells and all the signals, paracrine or autocrine that influence cell fate commitment. By using this model, we could identify and analyze two different molecules, TGFβ1 and WNT6, along with their cognate pathways that are involved in the endothelial commitment of ES cells. Other than for cell differentiation studies, the ES/PA6 co-culture system, thanks to its propensity to generate endothelium in response to precise exogenous signals, might be useful to unveil possible molecular targets to develop anti-angiogenesis therapies in cancer and for multi anti-angiogenic drugs screening.

## Supplementary Information

Below is the link to the electronic supplementary material.

Supplementary file1 (XLS 73 kb)

Supplementary file2 (PDF 7742 kb)

## Data Availability

The datasets generated during the current study are available in The Gene Expression Omnibus of the National Center for Biotechnology Information (Accession Number GSE164631). GSE 164631 is a SuperSeries composed by three different SubSeries: SubSeries GSE 164630 for time point 3 h; SubSeries GSE 164625 for time point 3 day; SubSeries 164629 for time point 7 day.
