# Supplementary material for: Role of TGFβ1 and WNT6 in FGF2 and BMP4-driven endothelial differentiation of murine embryonic stem cells
Source: Angiogenesis. 2021 Sep 3;25(1):113–28. doi: 10.1007/s10456-021-09815-4 (PMC8813801; doi:10.1007/s10456-021-09815-4)
Supplement: Supplementary file 2 — Supplementary file2 (PDF 7742 kb) [file 10456_2021_9815_MOESM2_ESM.pdf]

Supplementary Fig. 1

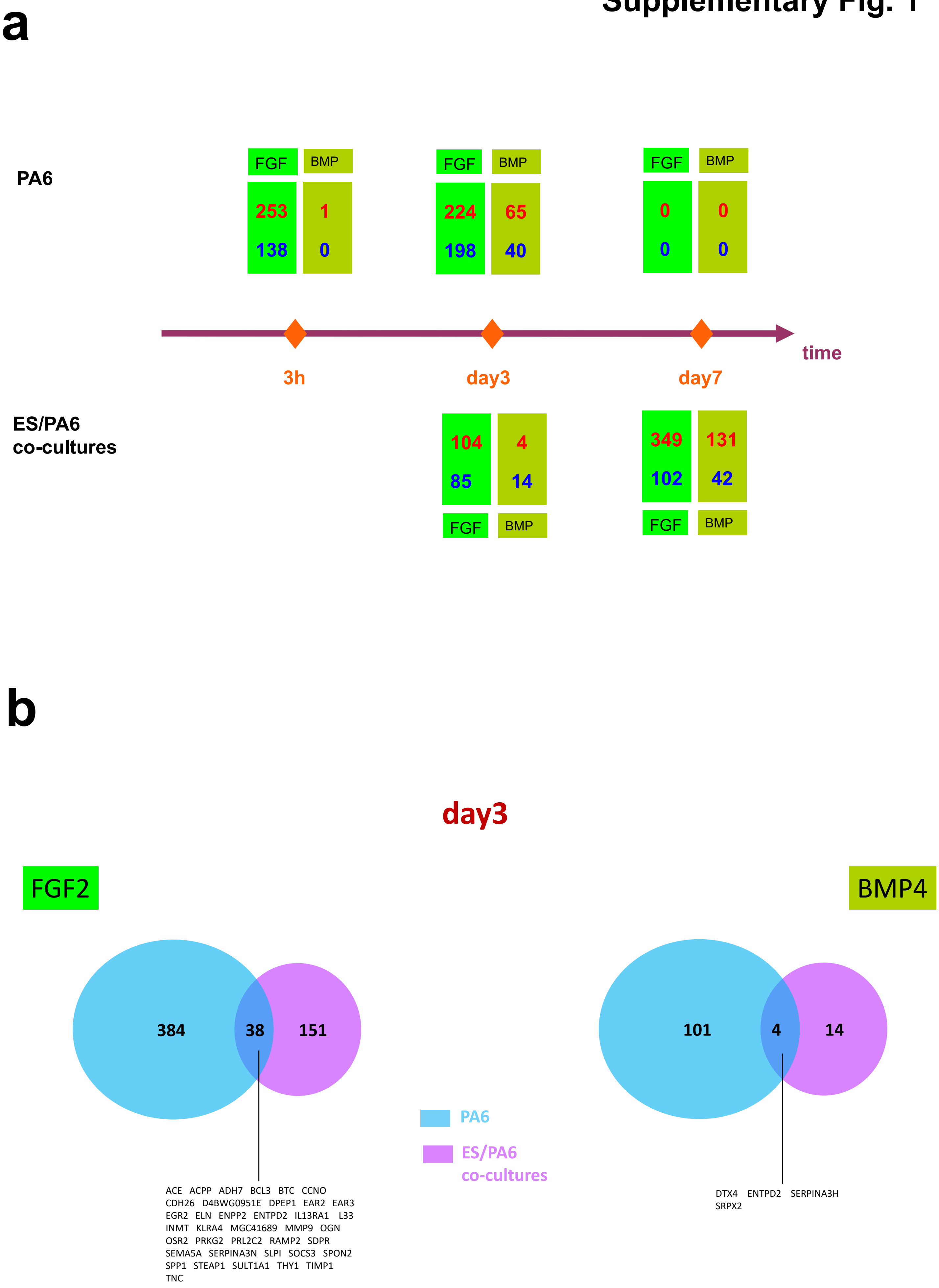

Supplementary Fig. 1 Analysis of gene expression of ES/PA6 co-cultures and PA6 mono-cultures upon FGF2 and BMP4 treatment.

(a) The pattern of gene expression of ES/PA6 co-cultures and PA6 mono-cultures upon –GFs condition and in presence of FGF2 or BMP4 was analyzed by microarray technique. A comparative transcriptome analysis of FGF2 and BMP4 treated cells versus -GFs condition was performed by LIMMA. The analysis spanned over 7 days of culture and comprised two time points for ES/PA6 co-cultures, 3 days and 7 days respectively, and three time points for pure cultures of PA6 cells. Indeed, other than 3 days and 7 days, pure culture of PA6 cells were analyzed after 3 hours of treatment to evidence the early responsive genes that were immediately activated in response to FGF2 and BMP4. For each time point and each treatment up-regulated genes are represented in red and the down-regulated genes are represented in blue. **(b)** After 3 days of culture the genes regulated by FGF2 and BMP4 shared by both ES/PA6 co-cultures and pure cultures of PA6 cells, as well as those uniquely regulated in ES/PA6 co-cultures or in PA6 cells alone, were represented by a Venn diagram

Supplementary Fig. 2

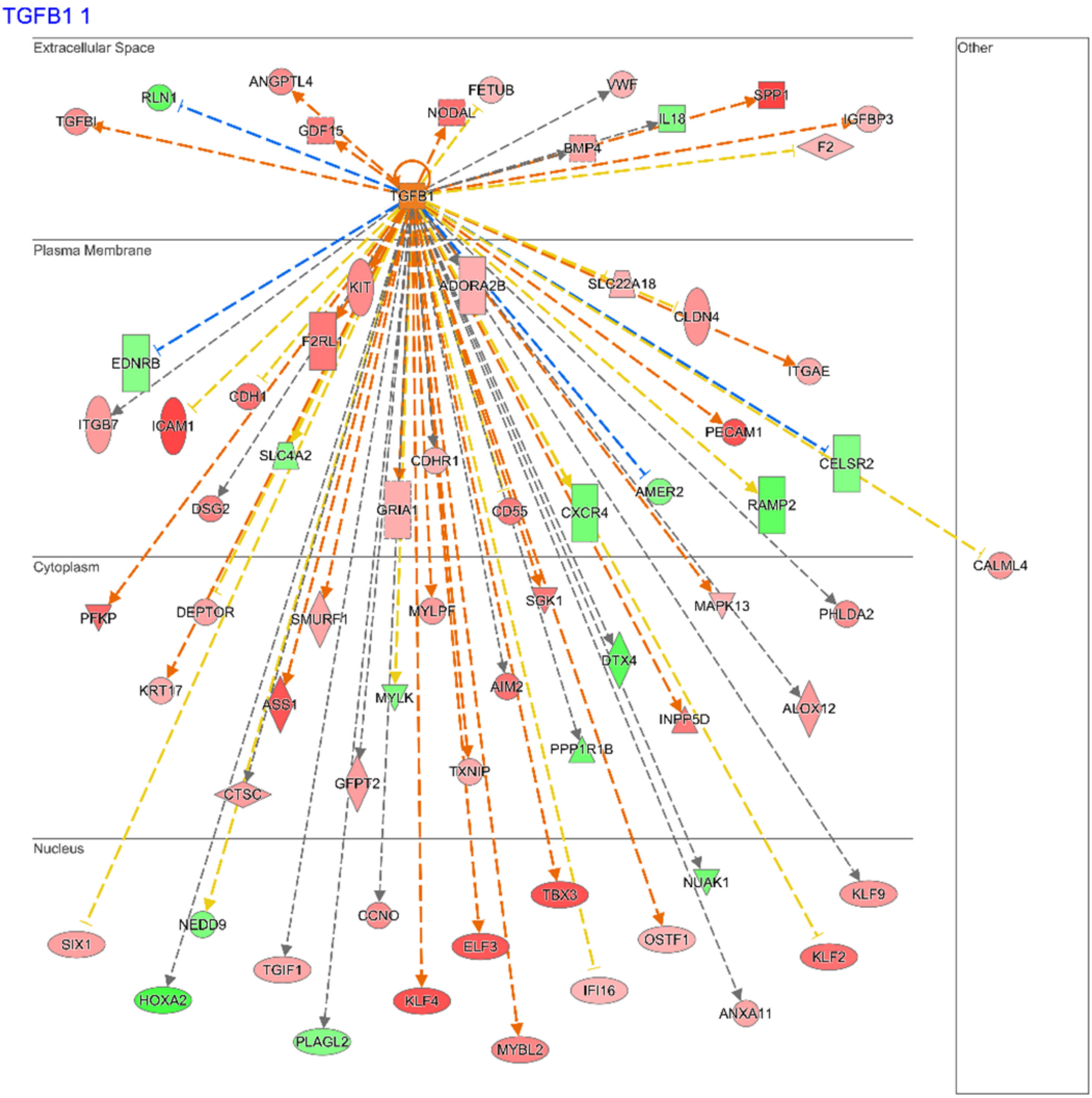

Supplementary Fig. 2 Identification of TGFβ1 and of its related molecules among the genes differentially expressed by ES/PA6 co-cultures after 7 days of treatment with FGF2.

The analysis of the differentially expressed genes by ES/PA6 co-cultures by IPA Ingenuity software identified TGFβ1 as the most represented upstream regulator activated in response to FGF2 after 7 days of treatment. TGFβ1, located in the center of the drawing, is connected to each of its 68 indirect interactors through dashed lines of different colors: brown indicates a predicted relationship that leads to activation; blue indicates a predicted relationship that leads to inhibition; yellow indicates findings inconsistent with state of downstream molecule; gray indicates a relationship whose effect is not predicted. Dashed lines terminating with an arrow stand for activation while the ones terminating with a bar stand for inhibition. TGFβ1 and its indirect interactors are represented in red if up-regulated and in green if down-regulated with different shade of color depending on the grade of modulation. TGFβ1 and its indirect interactors are distributed in the different cellular compartments

Supplementary Fig. 3

Wnt/β-catenin Signaling : day7.limma_BMP_vs_no_GF.NO_PA6.selected_1.Ingenuity : Expr Log Ratio

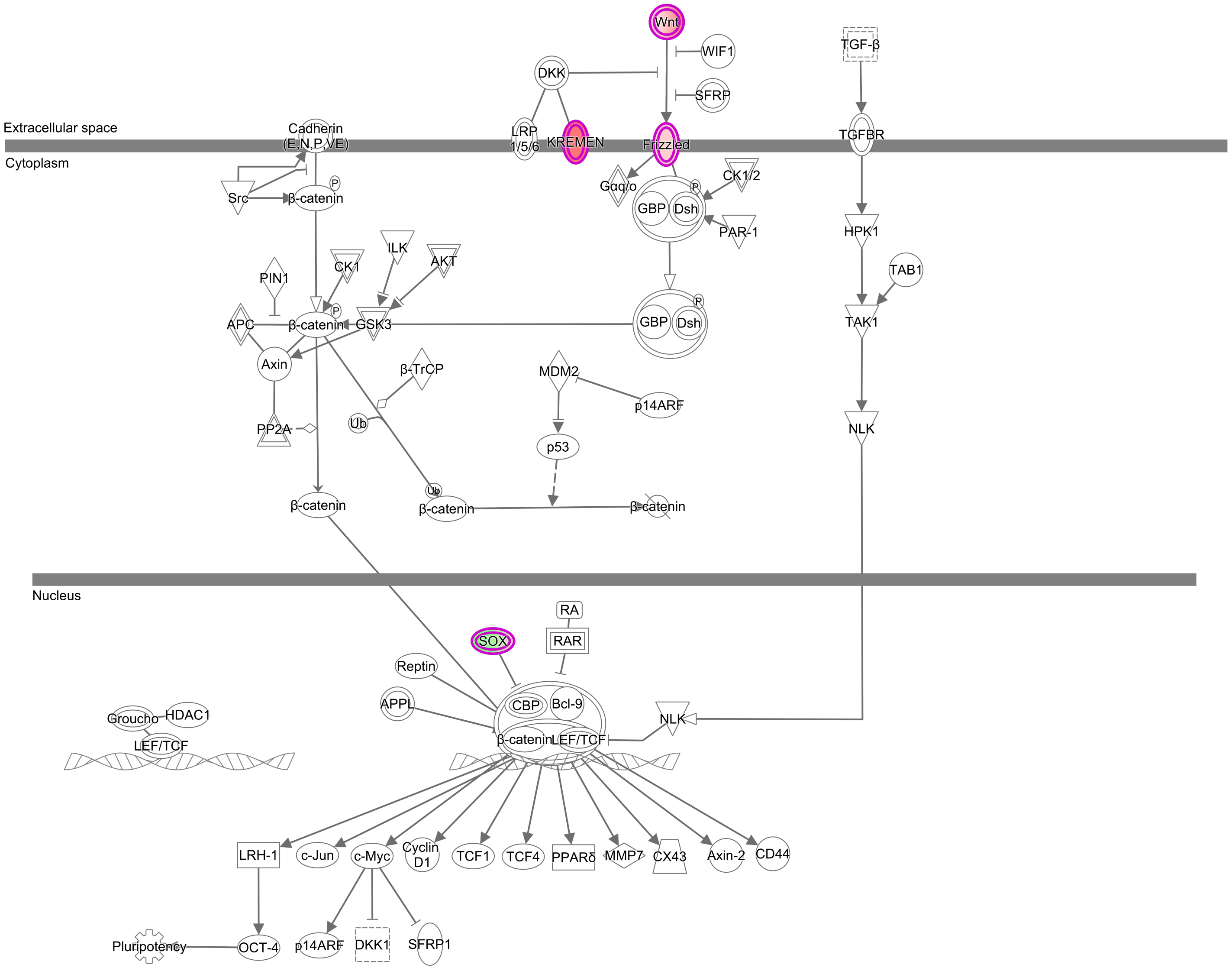

© 2000-2021 QIAGEN. All rights reserved.

Supplementary Fig. 3 Identification of WNT pathway as the most represented molecular mechanism activated by BMP4 in ES/PA6 co-cultures after 7 days of treatment.

The analysis of the differentially expressed genes by ES/PA6 co-cultures by IPA Ingenuity software identified WNT signaling as the most probable pathway activated by BMP4. All the molecules belonging to the WNT pathway are represented as uncolored boxes distributed in the different cellular compartments. The WNTs ligand and WNT-related molecules detected among the genes differentially expressed of ES/PA6 co-cultures are colored in red if up-regulated and in green if down-regulated. The double coloration of SOX box (green core and red border) stands for the down-regulation of SOX21 (green core) and the up-regulation of SOX17 (red border)

Supplementary Fig. 4

a

b

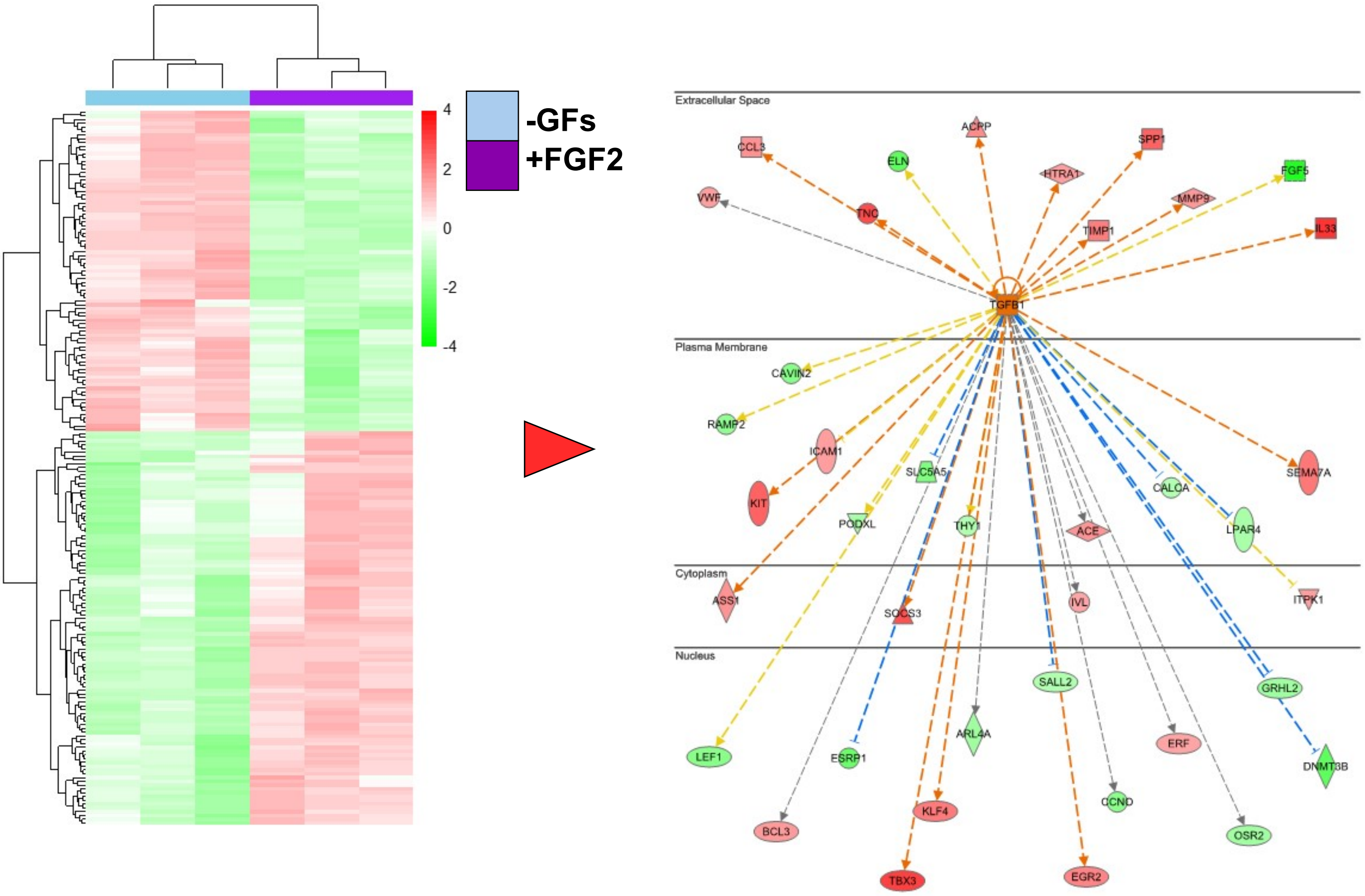

c

TGFβ1 related molecules			
Up-regulated		*Down-regulated*	
**Related to endothelial phenotype**	**Not related to endothelial phenotype**	**Related to endothelial phenotype**	**Not related to endothelial phenotype**
ACE	ACPP	CALCA	ARL4A
ASS1	EGR2	DNMT3B	CCND
BCL3	ERF	GRHL2	ESRP1
CCL3	IVL	LPAR4	OSR2
HTRA1			SALL2
IL33			SLCA5
KIT			
KLF4			
MMP9			
SEMA7A			
SOCS3			
SPP1			
TBX3			
TIMP1			
TNC			
VWF			

Supplementary Fig. 4 Analysis of gene expression of ES/PA6 co-cultures after 3 days of incubation with FGF2.

The pattern of gene expression of ES/PA6 co-cultures upon –GFs condition and FGF2 stimulation was examined by microarray technique. A comparative transcriptome analysis of FGF2 treated cells versus -GFs condition was performed by LIMMA and the results were visualized by heat map **(a)**. Red: up-regulated genes; green: down-regulated genes. **(b)** IPA Ingenuity analysis of up- and down-regulated genes identified TGFβ1 as one of the most represented upstream regulators along with 39 indirect interactors. TGFβ1, located in the center of the drawing, is connected to each of its interactors through dashed lines of different colors: brown indicates a predicted relationship that leads to activation; blue indicates a predicted relationship that leads to inhibition; yellow indicates findings inconsistent with state of downstream molecule; gray indicates a relationship whose effect is not predicted. Dashed lines terminating with an arrow stand for activation while the ones terminating with a bar stand for inhibition. TGFβ1 and its indirect interactors are represented in red if up-regulated and in green if down-regulated with different shade of color depending on the grade of modulation. The subcellular distribution is evidenced. **(c)** Those interactors that relate to TGFβ1 with consistency are listed accordingly to their modulation (up-regulated or down-regulated) and subdivided into two groups according to their relation with the observed endothelial phenotype

Supplementary Fig. 5

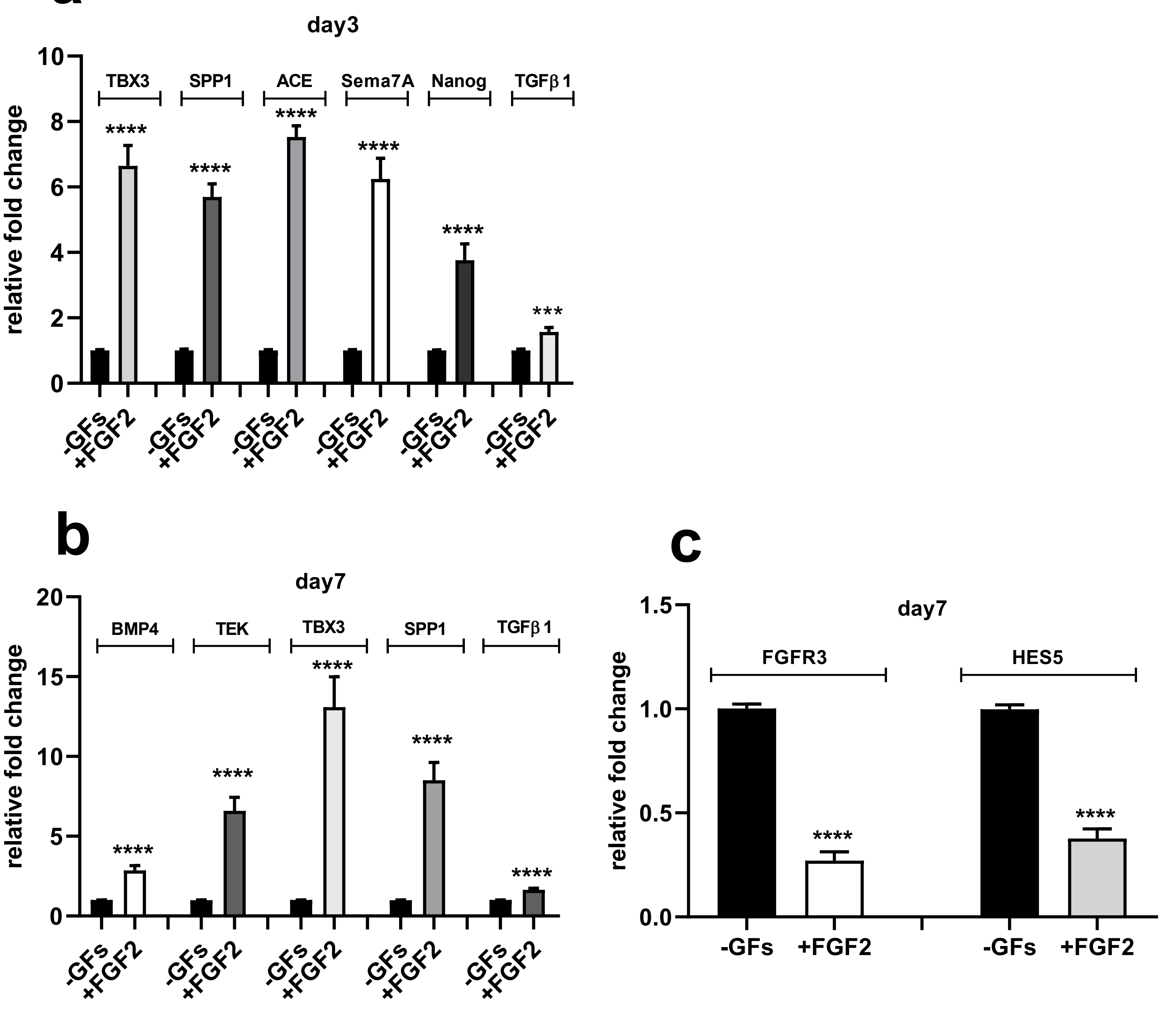

Supplementary Fig. 5 Validation by real time PCR of the modulation of the expression of some genes found to be differentially expressed by microarrays in ES/PA6 co-cultures treated with FGF2.

(**a**) The levels of expression of TBX3, SPP1, ACE, Sema7A, Nanog and TGFβ1 genes were evaluated by real time PCR of ES/PA6 co-cultures grown for 3 days in presence of FGF2. The data were normalized to the housekeeping gene TBP and expressed as relative fold change over the levels of expression detected in -GFs co-cultures ($n = 3$, mean ± SEM; ***$p = 0.001$, ****$p < 0.0001$). **(b)** The levels of expression of BMP4, TEK, TBX3, SPP1 and TGFβ1 genes were evaluated by real time PCR of ES/PA6 co-cultures grown for 7 days in presence of FGF2. After normalization to the housekeeping gene TBP, data were expressed as relative fold change over the levels of expression detected in -GFs co-cultures ($n = 4$, mean ± SEM; ****$p < 0.0001$). **(c)** The down-regulation of the expression of FGFR3 and HES5 genes was confirmed by real time PCR of ES/PA6 co-cultures grown for 7 days upon FGF2 stimulation. The data were normalized to the housekeeping gene TBP and expressed as relative fold change over the levels of expression detected in -GFs co-cultures ($n = 4$, mean ± SEM; ****$p < 0.0001$)

Supplementary Fig. 6

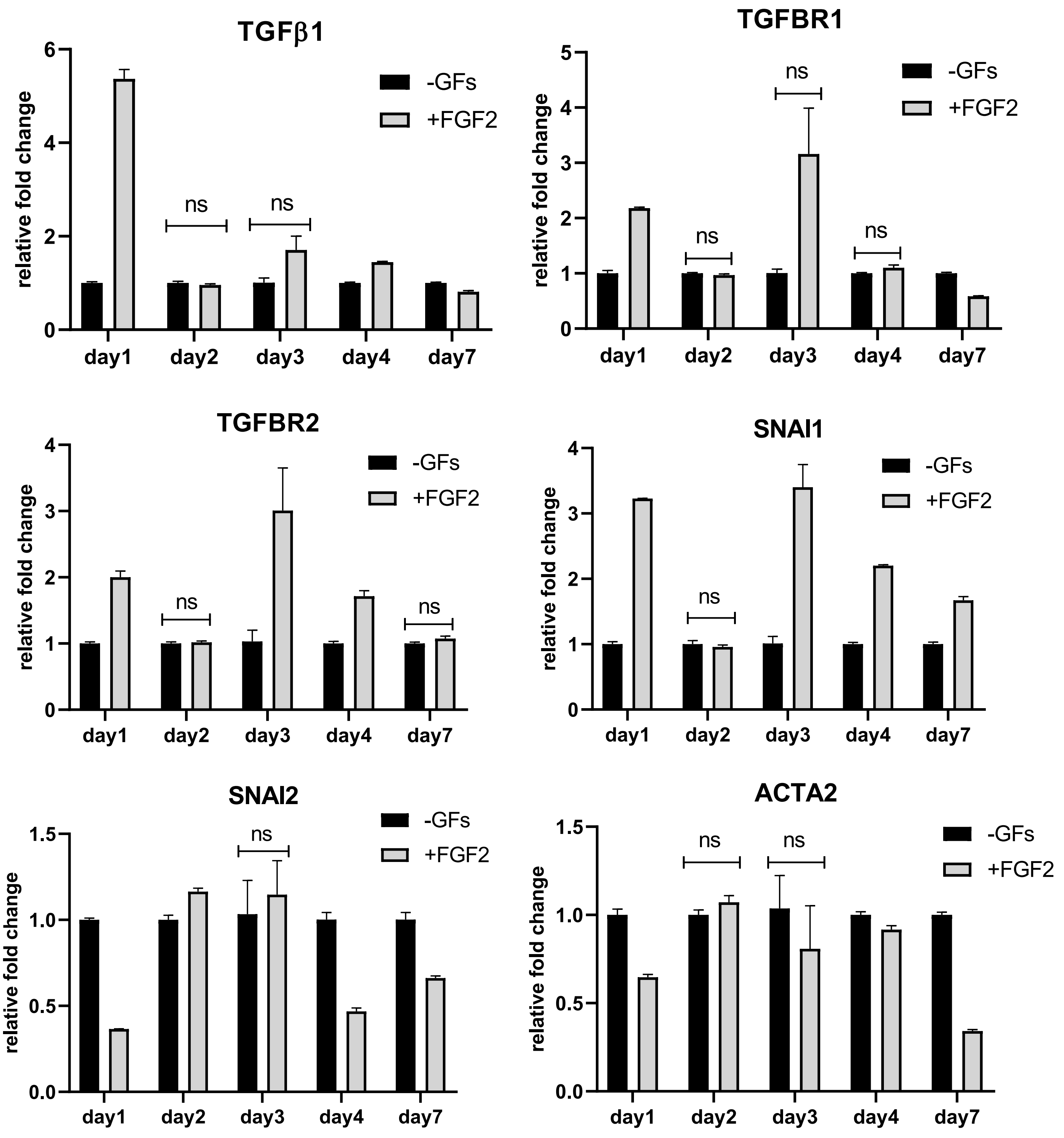

Supplementary Fig. 6 Analysis by real time PCR of the modulation of the expression of TGFβ1, TGFβ1-receptors and TGFβ1-target genes in ES/PA6 co-cultures treated with FGF2.

The levels of expression of TGFβ1, TGFBR1, TGFBR2, SNAI1, SNAI2 and ACTA2 were analyzed by real time PCR in ES/PA6 co-cultures at different time points and upon FGF2 treatment. For each gene, at each time point, the data were normalized to the housekeeping gene TBP and expressed as relative fold change over the levels of expression detected in the correspondent -GFs co-cultures (ns = not significant and $p < 0.05$ if not specified)

Supplementary Fig. 7

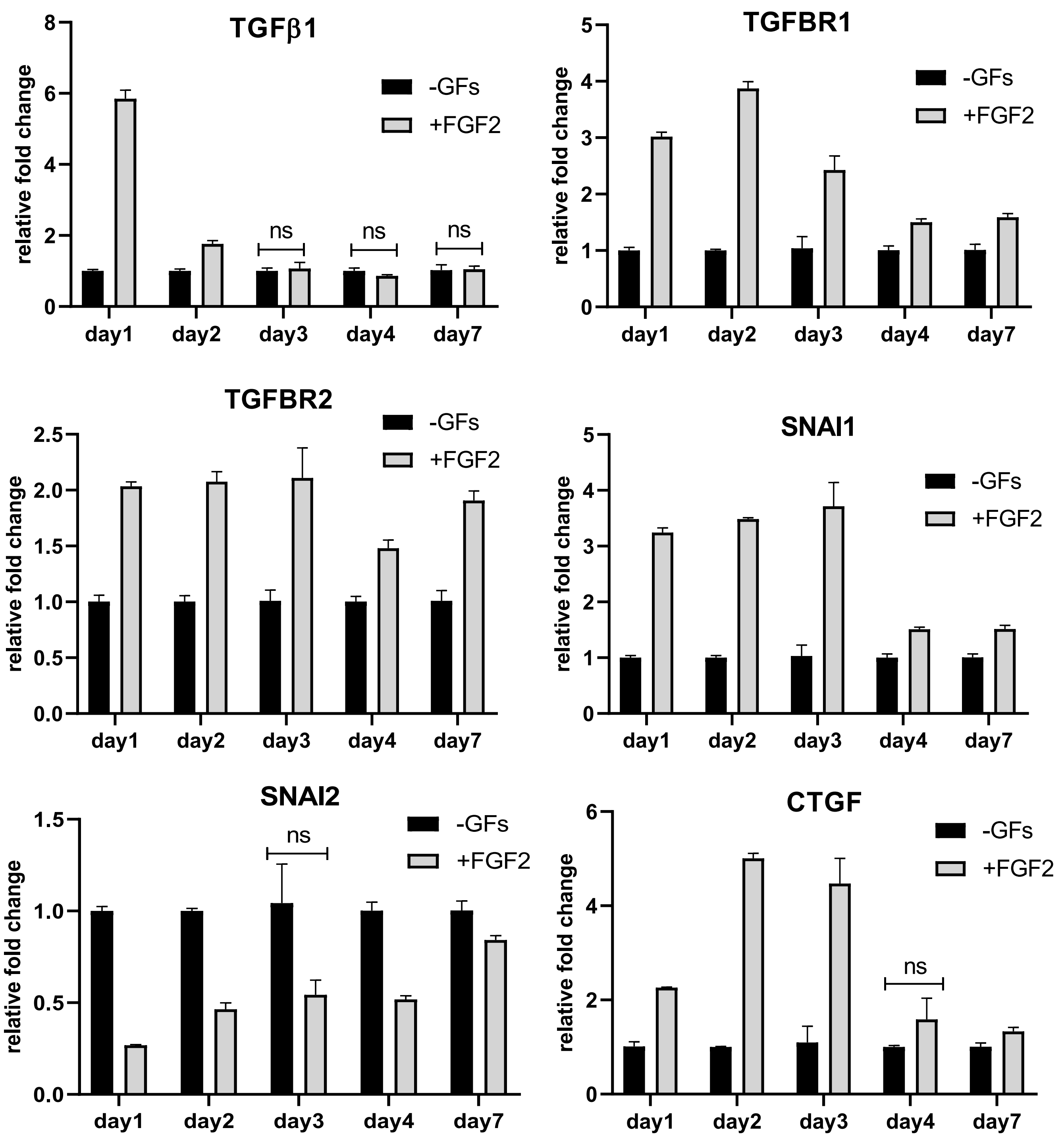

Supplementary Fig.7 Analysis by real time PCR of the modulation of the expression of TGFβ1, TGFβ1-receptors and TGFβ1-target genes in pure cultures of PA6 cells treated with FGF2.

The levels of expression of TGFβ1, TGFBR1, TGFBR2, SNAI1, SNAI2 and CTGF were analyzed by real time PCR in pure cultures of PA6 cells at different time points and upon FGF2 treatment. For each gene, at each time point, the data were normalized to the housekeeping gene TBP and expressed as relative fold change over the levels of expression detected in the correspondent -GFs cultures (ns = not significant and $p < 0.05$ if not specified)

Supplementary Fig. 8

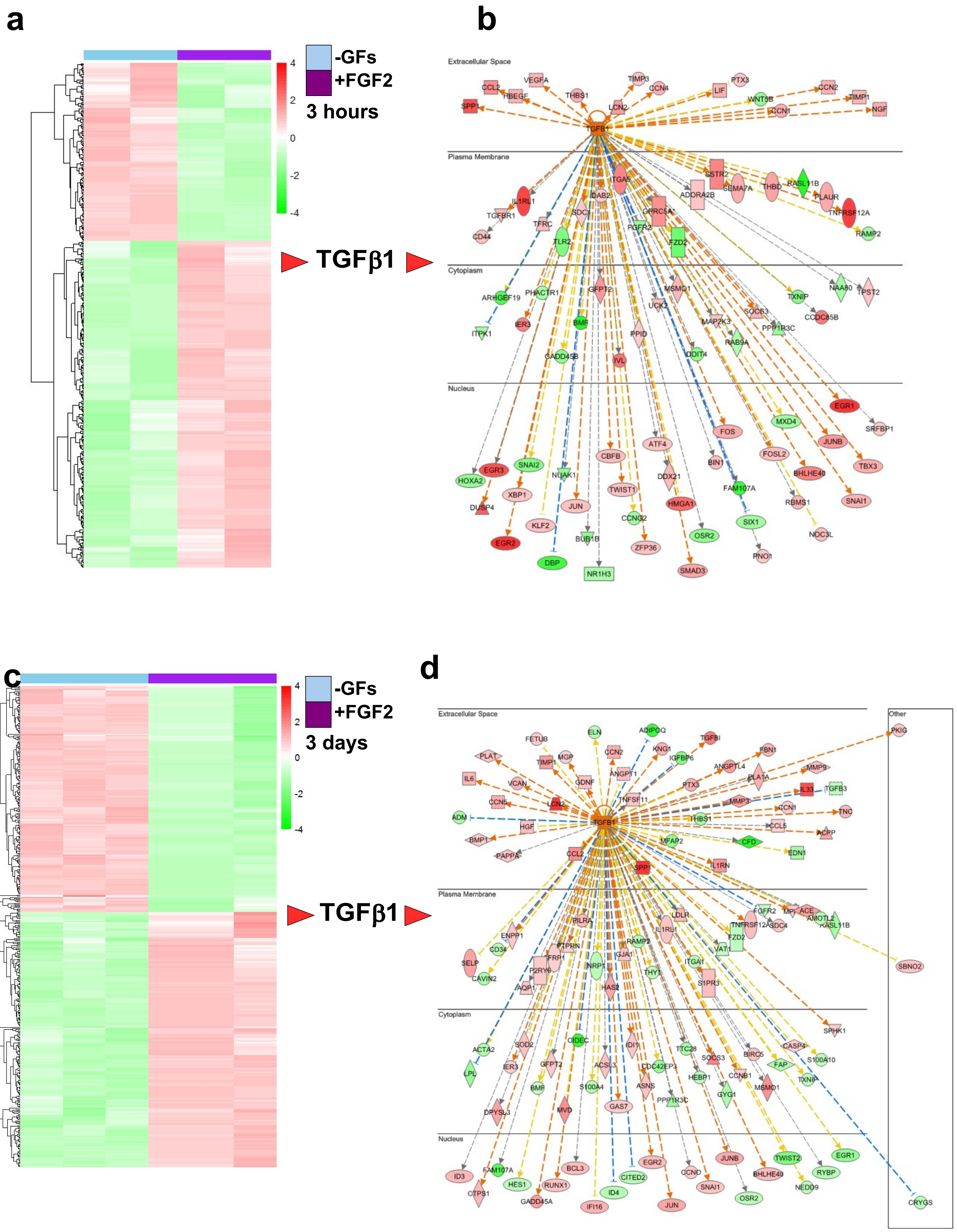

Supplementary Fig. 8 Analysis of gene expression of PA6 pure cultures after 3 hours and 3 days of treatment with FGF2.

(a) A gene expression analysis by microarray technique was performed on pure cultures of PA6 cells stimulated for 3 hours with FGF2. A comparative transcriptome analysis of FGF2 treated cells versus -GFs condition was performed by LIMMA and the results were visualized by heat map. Red: up-regulated genes; green: down-regulated genes. **(b)** IPA Ingenuity analysis of up- and down-regulated genes identified TGFβ1 as one of the most represented upstream regulators along with 91 indirect interactors. TGFβ1, located in the center of the drawing, is connected to each of its indirect interactors through dashed lines of different colors: brown indicates a predicted relationship that leads to activation; blue indicates a predicted relationship that leads to inhibition; yellow indicates findings inconsistent with state of downstream molecule; gray indicates a relationship whose effect is not predicted. Dashed lines terminating with an arrow stand for activation while the ones terminating with a bar stand for inhibition. TGFβ1 and its indirect interactors are represented in red if up-regulated and in green if down-regulated with different shade of color depending on the grade of modulation. The subcellular distribution is evidenced. **(c)** Heat map showing the up-regulated genes (in red) and the down-regulated genes (in green) identified by LIMMA comparative transcriptome analysis of FGF2-treated PA6 cells versus -GFs condition after 3 days of culture. **(d)** TGFβ1, located in the center of the drawing, was identified by IPA Ingenuity analysis as the most represented upstream regulator. 119 TGFβ1 indirect interactors are connected to TGFβ1 through dashed lines of different colors: for code colors see above (legend 8b). The subcellular distribution is evidenced

Supplementary Fig. 9

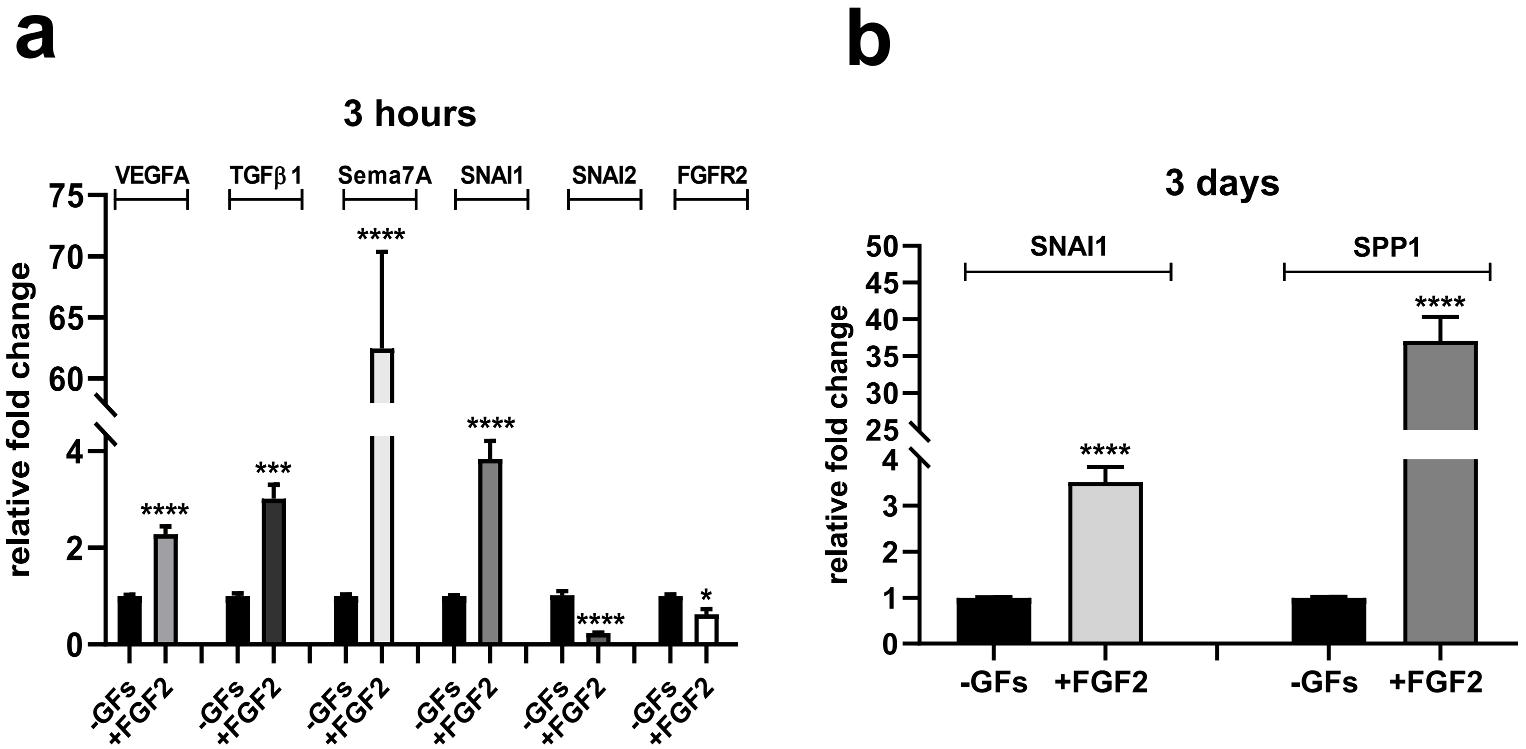

Supplementary Fig. 9 Validation by real time PCR of the modulation of the expression of some genes found to be differentially expressed by microarray in PA6 cells after 3 hours and 3 days of stimulation with FGF2.

(a) The levels of expression of VEGFA, TGFβ1, Sema 7A, SNAI1, SNAI2, FGFR2 genes were evaluated by real time PCR in PA6 pure cultures incubated for 3 hours with FGF2. The data were normalized to the housekeeping gene TBP and expressed as relative fold change over the levels of expression detected in -GFs cultures (n = 3, mean ± SEM; *$p < 0.05$, ***$p = 0.001$, ****$p < 0.0001$). **(b)** The levels of expression of SNAI1 and SPP1 genes were quantified by real time PCR in PA6 pure cultures grown for 3 days in presence of FGF2. After normalization to the housekeeping gene TBP, data were expressed as relative fold change over the levels of expression detected in -GFs cultures (n = 3, mean ± SEM; ****$p < 0.0001$)

Supplementary Fig. 10

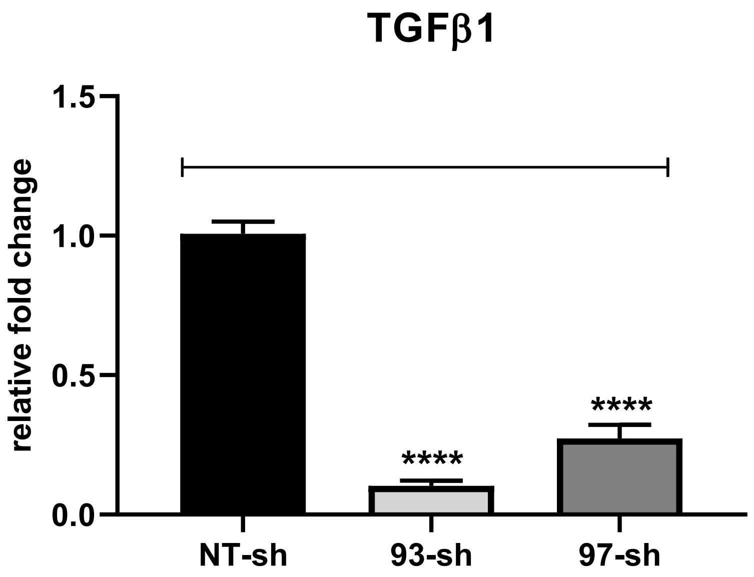

Supplementary Fig. 10 Efficiency of TGFβ1 silencing of PA6 cells.

PA6 cells were infected with lentiviral vectors carrying TGFβ1−specific shRNAs: 93-sh and 97-sh. A non transducing sh (NT-sh) was used as control. The silencing effect of shRNAs was confirmed by measuring the expression of TGFβ1-mRNA by real time PCR. Data were normalized to the housekeeping gene TBP and expressed as relative fold change over the levels of expression detected in NT-sh transduced cells (n = 3, mean ± SEM; ****$p < 0.0001$)

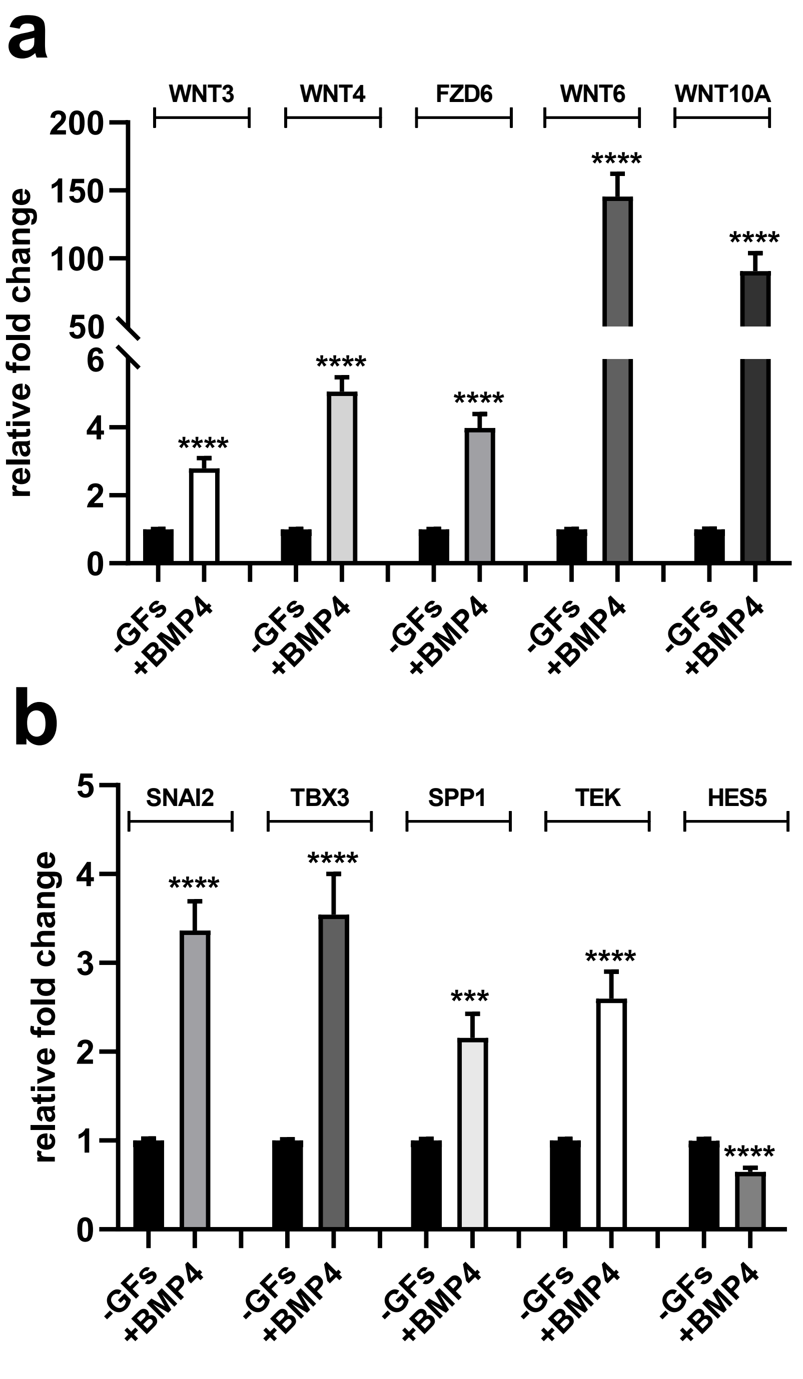

Supplementary Fig. 11 Validation by real time PCR of the modulation of the expression of some genes found to be differentially expressed by microarray in ES/PA6 co-cultures after 7 days of culture and upon BMP4 treatment.

(a) The levels of expression of WNT3, WNT4, FZD6, WNT6 and WNT10A genes were evaluated by real time PCR in ES/PA6 co-cultures stimulated with BMP4 and grown for 7 days. Data were normalized to the house keeping gene TBP and expressed as relative fold change over the levels of expression detected in -GFs co-cultures (n = 4, mean ± SEM; ****$p < 0.0001$). **(b)** The levels of expression of SNAI2, TBX3, SPP1, TEK and HES5 genes were measured by real time PCR in ES/PA6 co-cultures stimulated with BMP4 and grown for 7 days. After normalization to the housekeeping gene TBP, data were expressed as relative fold change over the levels of expression detected in -GFs co-cultures (n = 4, mean ± SEM; ***$p = 0.001$, ****$p < 0.0001$)

Supplementary Fig. 12

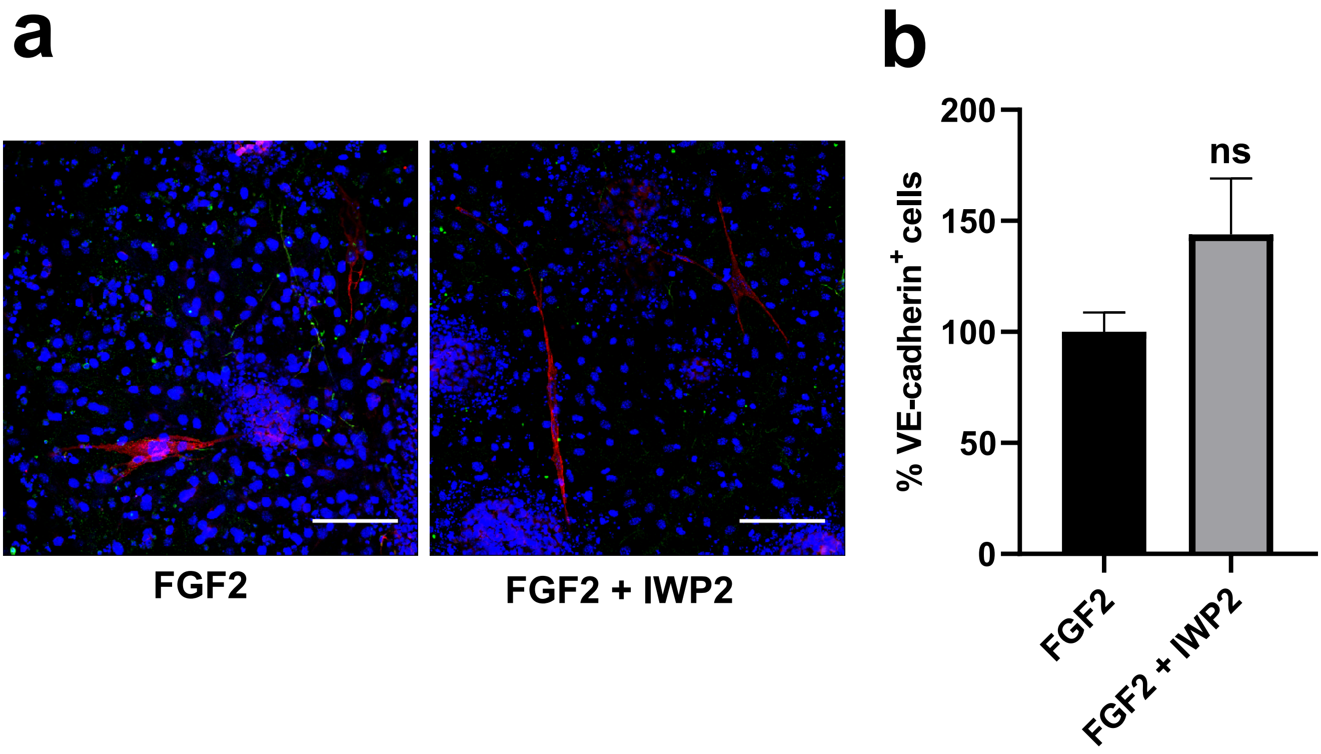

Supplementary Fig. 12 FGF2-induced endothelial differentiation of ES/PA6 co-cultures upon WNT pathway inhibition.

ES/PA6 co-cultures were grown for 7 days upon FGF2 treatment in absence or presence of the WNT inhibitor IWP2. **(a)** Blocking WNT activity didn't affect the capacity of FGF2 to induce endothelial differentiation as shown by the immunofluorescent staining performed with the anti-VE-cadherin antibody to detect endothelial cells (in red). Nuclei were evidenced in blue by DAPI staining. Scale bars: 50 µm. **(b)** The effect exerted by IPW2 on FGF2-induced endothelial differentiation was quantified by measuring the area occupied by VE-cadherin$^+$ cells. The data derived from ES/PA6 co-cultures in presence of IWP2 were normalized against those obtained in absence of IWP2 and expressed as percentage of VE-cadherin$^+$ cells (n = 3, mean ± SEM; ns = not significant)

Supplementary Fig. 13

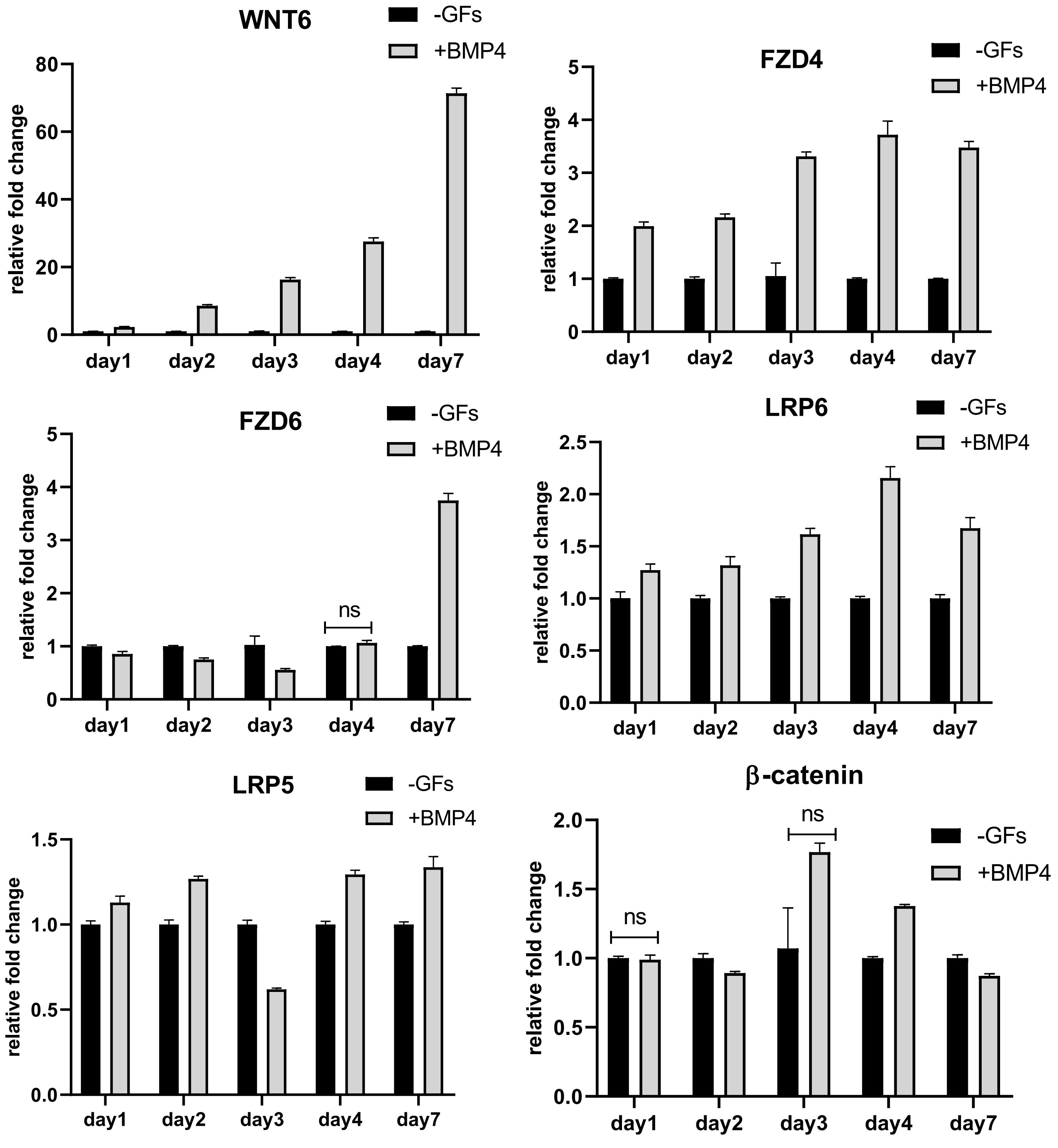

Supplementary Fig. 13 Analysis by real time PCR of the modulation of the expression of WNT6, WNT receptors and β-catenin genes in ES/PA6 co-cultures treated with BMP4.

The levels of expression of WNT6, FZD4, FZD6, LRP6, LRP5 and β-catenin were analyzed by real time PCR in ES/PA6 co-cultures at different time points upon BMP4 treatment. For each gene at each time point the data were normalized to the housekeeping gene TBP and expressed as relative fold change over the levels of expression detected in the correspondent -GFs co-cultures (ns = not significant and $p < 0.05$ if not specified)

Supplementary Fig. 14

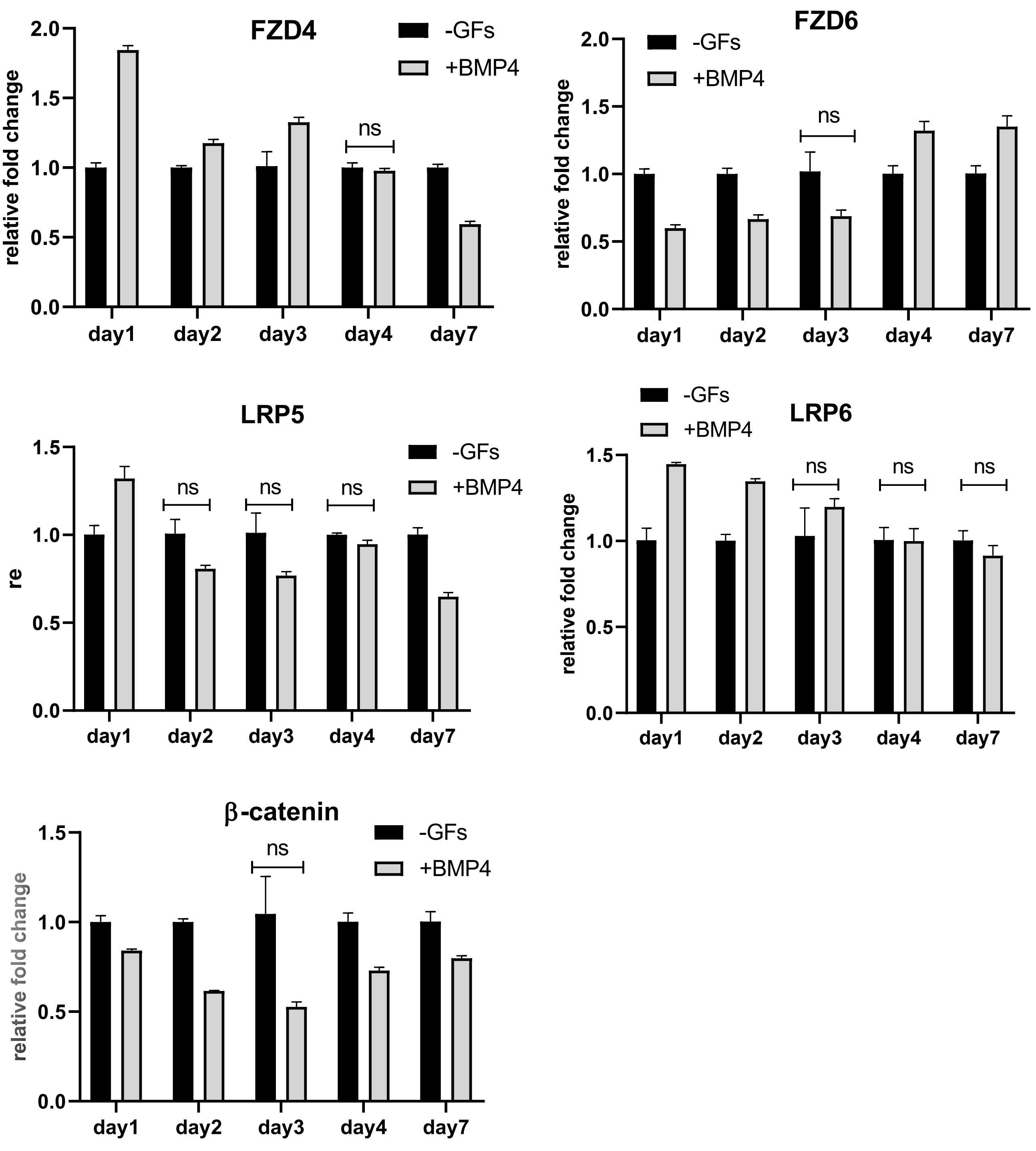

Supplementary Fig.14 Analysis by real time PCR of the modulation of the expression of WNT receptors and β-catenin genes in pure cultures of PA6 cells treated with BMP4.

The levels of expression of FZD4, FZD6, LRP5, LRP6 and β-catenin were analyzed by real time PCR in pure cultures of PA6 cells at different time points. For each gene at each time point the data were normalized to the housekeeping gene TBP and expressed as relative fold change over the levels of expression detected in the correspondent -GFs co-cultures (ns = not significant and $p < 0.05$ if not specified)

Supplementary Fig. 15

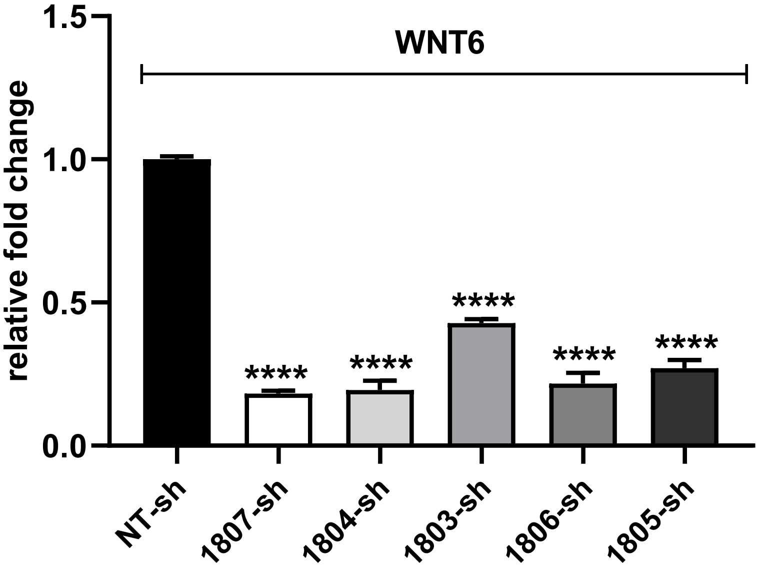

Supplementary Fig. 15 Efficiency of WNT6 gene silencing by lentiviral shRNAs.

ES/PA6 co-cultures were infected with WNT6-specific shRNAs carried by lentiviral vectors. A non-transducing shRNA (NT-sh) was used as control. After 7 days of culture, upon BMP4 treatment, total RNA was purified and analyzed to detect WNT6 expression by real time PCR. Data were normalized to the housekeeping gene TBP and expressed as relative fold change over the values obtained with -GFs co-cultures ($n = 3$, mean ± SEM; ****$p < 0.0001$)
